# Lipoic acid-engineered nanodroplets for multimodal imaging and enhanced sonodynamic therapy in melanoma treatment

**DOI:** 10.7150/thno.127554

**Published:** 2026-03-09

**Authors:** Ziyao Wang, Yongchao Yao, Ziyan Feng, Yulong Liao, Liyun Wang, Xueyang Xiao, Wenchuang Hu, Zhiyong Qian, Li Qiu

**Affiliations:** 1 Department of Ultrasound, West China Hospital, Sichuan University, Chengdu 610041, Sichuan, China.; 2 Department of Biotherapy, Cancer Center and State Key Laboratory of Biotherapy, West China Hospital, Sichuan University, Chengdu 610041, Sichuan, China.; 3 Precision Medicine Translational Research Center (PMTRC), West China Hospital, Sichuan University, Chengdu 610041, Sichuan, China.

**Keywords:** melanoma, lipoic acid, enhanced sonodynamic therapy, contrast-enhanced ultrasound imaging, photoacoustic imaging

## Abstract

**Rationale:**

Sonodynamic therapy (SDT) has emerged as a promising strategy for melanoma treatment because of its noninvasive nature, deep penetration and potential to activate antitumor immunity. However, the uncontrollable delivery and limited tumor accumulation of sonosensitizers often restrict SDT efficacy. And tumor cells tend to activate cytoprotective autophagy under SDT-induced oxidative stress, which may further compromise treatment outcomes. Developing an image-guided drug delivery system with the function of enhancing SDT efficacy offers a promising strategy for melanoma management.

**Methods:**

We developed a new ultrasound (US)-responsive lipoic acid-based nanodroplets loaded with perfluoropentane (PFP) and sonosensitizer Chlorin e6 (LA@P-Ce6 NDs). The physicochemical properties, acoustic droplet vaporization (ADV) behavior, and biosafety of LA@P-Ce6 NDs were systematically evaluated. Contrast-enhanced ultrasound (CEUS) and photoacoustic (PA) imaging were conducted both *in vitro* and *in vivo* to evaluate multimodal imaging ability. Antitumor effects and mechanisms of LA@P-Ce6 NDs under US irradiation were investigated both *in vitro* and *in vivo*, as well as their ability to activate antitumor immunity.

**Results:**

LA@P-Ce6 NDs exhibited excellent stability under physiological conditions and underwent effective ADV, enabling CEUS and PA imaging both *in vitro* and *in vivo*. Under therapeutic US, Ce6 induced strong ROS production and LA modulated autophagic flux, synergistically achieving enhanced oxidative damage at the irradiated site and promoting ICD. Our treatment showed significant antitumor ability and induced strong antitumor immune effect, which was able to suppress pulmonary metastasis.

**Conclusions:**

This study presented a multifunctional LA@P-Ce6 NDs system that enabled autophagy-modulated, CEUS/PA image-guided SDT. The integration of multimodal imaging and synergistic antitumor therapy offers a promising melanoma management method.

## Introduction

Melanoma is one of the most aggressive skin cancers, with over 330,000 new cases and 58,667 deaths worldwide in 2022 1. Due to population aging, increased ultraviolet (UV) radiation exposure, and tanning-related behaviors, the global incidence of cutaneous melanoma continues to rise, and the number of new cases is predicted to reach over 500,000 by 2040 2. Many early-stage melanoma patients can be cured by surgery 3, but treatment options for late-stage patients are limited and the efficiency is relatively unsatisfactory 4. Nowadays, immunotherapy and target therapy have become standard treatments for advanced patients 5. However, drug resistance and limited efficacy remain clinical challenges, resulting in the 5-year survival rate drops to just over 30% for advanced patients 6. This poor prognosis is largely attributed to treatment resistance driven by tumor heterogeneity 7, immune evasion, and the immunosuppressive tumor microenvironment (TME) 8. Tumor cell killing mediated by reactive oxygen species (ROS), as in photodynamic therapy (PDT) and sonodynamic therapy (SDT), has the potential to induce immunogenic cell death (ICD) 9, 10, during which the dead tumor cells can stimulate antigen-presenting cells and promote T cell priming, and ultimately amplify anti-tumor immunity 11. However, compared with near-infrared (NIR) light used in PDT, ultrasound (US) used in SDT exhibits significantly superior penetration ability, reaching several centimeters into tissues 12, 13, making it more suitable for treating advanced melanoma with local infiltration. US as a mechanical wave also offers more precise focus on target area and more complete triggering and release of drug *in situ* than NIR light 14, 15. Therefore, SDT with the advantages of non-invasiveness, deep tissue penetration and spatiotemporal controllability represents a more viable treatment for advanced melanoma than PDT. Chlorin e6 (Ce6), a second-generation porphyrin-based sonosensitizer, represents excellent safety and strong ROS generation, and is proven preclinically effective 16. However, the therapeutic efficacy of Ce6 is often compromised by poor water solubility, insufficient tumor accumulation, and nonspecific biodistribution 17, 18. Furthermore, SDT as a monotherapy may be inadequate to fully eradicate tumors, especially in immunosuppressive regions 19.

To address these limitations, a variety of drug delivery systems (DDSs), including liposomes, polymeric micelles, and inorganic nanoparticles, have been developed to enhance the bioavailability and tumor-specific accumulation of sonosensitizers 20. These carriers increase local drug concentration in tumor tissues via enhanced permeability and retention (EPR) effect or active targeting, boosting therapeutic efficacy while minimizing systemic exposure 21. Nevertheless, conventional DDSs face challenges. Heterogeneous EPR effects lead to undesired distribution 22, the lack of spatiotemporal control over drug release reduces treatment specificity 23, and the dense extracellular matrix hinders deep tumor penetration 24. These challenges highlight the need of new DDSs to address these problems. US-responsive microbubbles (MBs) provide a promising solution by combining drug delivery with real-time imaging guidance 25 and controlled drug release through US-mediated cavitation 26. US-mediated cavitation also increases vascular and cellular permeability, facilitating efficient drug transport via US-targeted microbubble destruction (UTMD) effect 27. Structurally, MBs consist of a gas core encapsulated by a stabilizing shell, which endow them with excellent echogenicity and high responsiveness to US stimulation 28. To improve systemic circulation and drug delivery efficiency, these US contrast agents have been progressively miniaturized to nanodroplets (NDs) with a liquid perfluorocarbon (PFC) core 29, 30. Upon US exposure, the liquid cores of NDs undergo acoustic droplet vaporization (ADV), transforming NDs into MBs in situ. Their nanoscale dimensions and their responsiveness to US enable them with the abilities including extravasation into tumor tissues, drug delivery visualization, spatiotemporally controlled activation, and on-demand drug release 31. Depending on their compositions, some drug-loaded NDs can exhibit both contrast-enhanced ultrasound (CEUS) and photoacoustic (PA) imaging capability 32. Collectively, these advantages make NDs highly attractive for theranostic applications, integrating multimodal imaging with precise, image-guided therapy.

Rational shell design is critical to optimize the functions of NDs. During ADV, the gas core has to expand to overcome the constraint of the shell to vaporize into a MB. The shell plays the role to maintain stability during systemic circulation yet allow efficient ADV under clinically safe US irradiation. Shell elasticity directly impacts ADV threshold, since NDs have to overcome intrinsic Laplace pressure and the constraint of the shells to expand. Overly rigid shells stabilize the droplets but require excessive acoustic energy to perform ADV and compromise NDs' echogenicity, and overly soft shells risk premature rupture and leakage 33. Precise modulation of shell elasticity is critical for balancing stability, imaging performance, and drug release efficiency. In this way NDs can be used in more diverse application scenarios. Recently, polymer-based shells have gained interest due to their tunable mechanical properties, crosslinking density, and chemical functionality, allowing fine control over ADV behavior and drug release 34.

Beyond serving as a passive carrier, the shell can also be designed as a therapeutic component. Inspired by the emerging concept of drug self-delivery systems (DSDSs), where active drug molecules self-assemble into polymers and function as both carrier and cargo 35, the shells of NDs are promising to be constructed by therapeutically active monomers. Such a design not only offers high drug loading capacity but also avoids carrier-related toxicity 36 and enables US-triggered drug release. It can also further minimize systemic toxicity. Such multifunctional shells are particularly appealing for combination strategies aimed at overcoming the limited therapeutic efficacy of SDT monotherapy. Addressing the cellular protective mechanisms caused by elevated ROS levels can enhance SDT efficacy. One promising approach is to modulate SDT-induced autophagy, a stress-adaptive process that can be activated under oxidative stress to protect tumor cells 37. Pharmacological autophagy flux inhibition can enhance ROS-mediated cytotoxicity and thereby amplifying ICD to trigger anti-tumor immunity 38, 39. However, systemic administration of autophagy inhibitors can induce off-target toxicity. Designing NDs with stimuli-responsive DSDS shells that remain therapeutically inert during circulation but disassemble in response to TME and US cavitation can ensure spatially controlled drug release, thereby improving therapeutic selectivity and synergistic efficacy of autophagy modulation combined with ROS-mediated therapy. This method can also help avoid systemic toxicity. In this way, a system that integrates an elastic, polymer-based and autophagy-modulating shell represents an exciting strategy for combining deep cellular penetration, visualized drug delivery, and minimal systemic toxicity, as well as synergistically maximizing SDT to achieve potent anti-melanoma efficacy.

**Figure 1:**
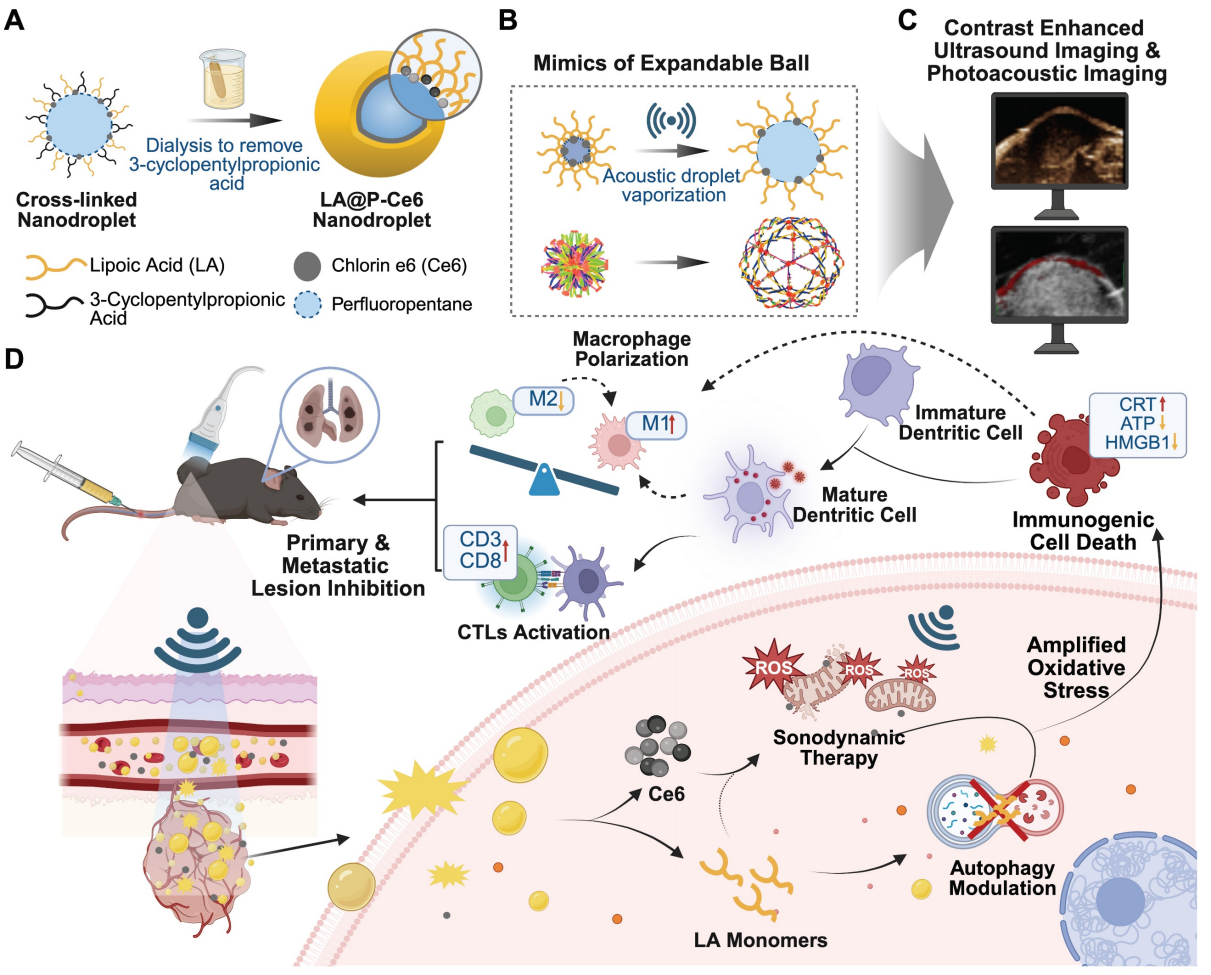
Schematic illustration of the design and therapeutic strategy of LA@P-Ce6 NDs. (A) Design strategy of LA@P-Ce6 NDs. (B) The ND shell gains elasticity by mimicking the structure of expandable balls. (C) Imaging ability of LA@P-Ce6 NDs. (D) Schematic illustration of the therapeutic strategy of LA@P-Ce6 NDs combining SDT and autophagy modulation for synergistic induction of anti-tumor immunity.

In this work, we present novel US-responsive DSDSs made up of crosslinked lipoic acid polymeric shells, perfluoropentane (PFP) cores and sonosensitizer Ce6 (LA@P-Ce6 NDs). The resulting NDs integrated CEUS/PA imaging with the combination of SDT and autophagy modulation for melanoma theranostics. LA is recognized to have a synergistic antitumor effect by regulating ROS level to induce tumor cell apoptosis with minimal impact on normal cells 40, 41, as well as influencing cell autophagic flux 42. As is shown in Figure 1A, to achieve the desired elasticity in LA shells, a temporary spacer was introduced to elongate the disulfide linkages of LA monomers during polymerization, allowing the shell to exhibit a flexible, elastic network once the spacer was removed. This design was the mimic of the elastic network of the expandable ball (Figure 1B), which ensures both mechanical stability in circulation and rapid acoustic responsiveness under mild US exposure. Before entering tumor cells, LA stayed polymerized in LA@P-Ce6 NDs to ensure circulation biosafety. Based on previous studies, glutathione (GSH) together with US is expected to trigger the cleavage of disulfide bonds in LA shells 43, 44. Upon reaching the TME where the GSH level is elevated 45, 46, LA@P-Ce6 could release LA monomers and Ce6. LA subsequently exerted its pharmacological activity by modulating ROS-induced cytoprotective autophagy, thereby enhancing Ce6-mediated SDT. Meanwhile, PFP vaporization generated enhanced US contrast, and Ce6 provided a PA signal, together allowing real-time monitoring of NDs accumulation and activation (Figure 1C). In this multifunctional LA@P-Ce6 NDs, PFP and Ce6 provided multimodal imaging, and the spatiotemporally controlled release of LA monomers and activation of Ce6 produced amplified oxidative stress, modulated autophagy, and promoted ICD. These therapeutic effects together induced a systemic immune response and suppressed lung metastasis (Figure 1D), and this design offered a promising strategy for precise theranostics in advanced melanoma.

## Methods

### Materials

(R)-(+)-Lipoic acid (LA) and chlorin e6(Ce6) were purchased from Tansoole. PFP was purchased from Aladdin. Singlet oxygen sensor green (SOSG) was purchased from Thermo Fisher Scientific Inc. Calcein AM/Propidium Iodide (PI) kit were obtained from Yeasen Biotech. Annexin V Cell Apoptosis Analysis kit and ATP Assay Kit were purchased from Beyotime Biotech. 2',7'-Dichlorofluorescein diacetate (DCFH-DA) was purchased from Sigma-Aldrich Co., Ltd. Calreticulin Rabbit mAb and HMGB1 Rabbit mAb were purchased from Abclonal Technology. The tandem mCherry-EGFP-LC3 adenovirus was purchased from Hanbio Biotechnology. LC3B (E5Q2K) Mouse mAb, CD3 Rabbit mAb, CD4 Rabbit mAb and CD8-alpha XP Rabbit mAb were purchased from Cell Signaling Technology, Inc. Opal Fluorophore Reagent Packs were purchased from Akoya Biosciences. All fluorescently-labeled antibodies for flow cytometric analysis of immune cells were obtained from BioLegend, Inc.

Murine melanoma cancer B16F10 cell and human umbilical vein endothelial cell (HUVEC) were obtained from American Type Culture Collection (ATCC). RPMI-1640, penicillin/streptomycin, and pancreatin were purchased from Thermo Fisher Scientific. C57BL/6 mice were obtained from Huafukang Biotechnology Co., Ltd. Animal study was approved by the Animal Ethics Committee of the West China Hospital, Sichuan University, Chengdu, China (No. 20231110005).

### Synthesis of LA@P-Ce6 NDs

LA@P-Ce6 NDs were synthesized using a modified oil-in-water emulsion method. Briefly, pentaerythritol tetra (3-mercaptopropionate) (25 mg, 0.05 mmol) and Ce6 (30 mg, 0.05 mmol) were dissolved in a mixed solvent of PFP and DMSO (1:1 v/v, 200 μL). This organic phase was then added dropwise into an aqueous solution (5 mL) containing lipoic acid sodium (228 mg, 1 mmol) and 3-cyclopentylpropionic acid sodium (41 mg, 0.25 mmol), and the mixture was subjected to ultrasonication for 30 min. The emulsion was then dialyzed (1.0 kDa MWCO) for 48 h to obtain the cross-linked LA@P-Ce6 NDs. The Ce6 loading capacity of the NDs was quantified by UV-Vis spectroscopy. LA@P NDs with LA shells and PFP cores were synthesized using the same procedure, except that Ce6 was not added during the synthesis, to serve as non-drug-loaded control NDs.

### Characterization of NDs

The stability of NDs (LA@P NDs and LA@P-Ce6 NDs) were evaluated in FBS and PBS environments. The NDs were incubated with 10% FBS (v/v) or PBS to assess their *in vitro* FBS stability. 9.0 mL NDs (1.0 mg/mL) was diluted in 1.0 mL FBS solution or PBS solution and incubated at 37 °C. The sizes and zeta potentials of the NDs were assessed using DLS (Malvern Zetasizer Nano ZS90) at various time points during 48 h of incubation time.

The NDs were observed with a transmission electron microscope (TEM) to monitor their sizes and morphology features. The ROS generation capability of LA@P-Ce6 NDs under US irradiation (3 W/cm^2^, 1 MHz, 10% duty cycle, 1 min) was assessed using a SOSG fluorescent probe.

### Cell culture

B16F10 cells and HUVECs were cultured in RPMI 1640 medium supplemented with 10% FBS, penicillin (100 U/mL), and streptomycin (100 μg/mL). The cells were maintained in a humidified incubator at 37 °C with 5% CO_2_.

### Cellular uptake assessment

B16F10 cells were inoculated into 24-well plates overnight, followed by the addition of NDs to each well and US treatment. A fluorescent microscope was used to observe Ce6's intracellular fluorescence at various time points.

### ROS detection

The ROS level were detected by DCFH-DA after different treatments. B16F10 cells were inoculated onto 24-well plates overnight, then treatments were administered to cells and cells were incubated overnight. Following a 30 min incubation period, DCFH-DA was employed as a ROS-detection fluorescence probe to each group of cells. Following PBS washing, intracellular fluorescence was observed using an Olympus IX 83 microscope and Cytoflex flow cytometer (Beckman Coulter, USA).

### Autophagy evaluation

After different treatments, B16F10 cells were lysed to harvest protein. Protein concentrations were assessed by BCA assay, and samples were loaded for SDS-PAGE and transferred to PVDF membranes. The membranes were blocked with blocking kit, followed by incubation with primary antibodies. After washing, the membranes were incubated with their corresponding secondary antibodies. The ECL Prime Western Blotting Detection Reagent (BIO-RAD, USA) was used to display the protein bands.

B16F10 cells were infected with an adenoviral mCherry-EGFP-LC3 reporter to visualize autophagic flux. After the cells successfully expressed fluorescence, they were treated with different treatments. The distribution patterns of mCherry and EGFP fluorescence signals were observed using an Olympus IX 83 microscope.

### Cell viability and apoptosis assay

CCK-8 assay was used to assess cell viability. B16F10 and HUVEC cells were added in a 96-well plate at a density of 10^4^ cells per well. After being processed with different treatments for 48 h, CCK-8 reagent was added at the end of the treatment and incubated for 2 h at 37 °C. The absorbance at 450 nm was measured using a microplate reader.

The live/dead assay was performed to visually assess cell viability post-treatment. B16F10 cells were cultured on cover slips and treated. Then a live/dead assay was performed according to the manufacturer's protocol, and the cells were monitored using an Olympus IX 83 microscope.

Following 48 h of different treatments and standard culture conditions, cells were harvested for flow cytometry analysis. For the apoptosis detection, cells were resuspended in buffer and incubated with Annexin V-FITC and PI. After dying, all samples were analyzed using Cytoflex flow cytometer.

### Immunofluorescence

After treatment, B16F10 cells on cover slips were fixed, permeabilized, and blocked. Primary antibodies were incubated overnight, followed by fluorescent secondary antibodies. After dying the nuclei with DAPI, the slides were sent for imaging. Fluorescence intensity and location features were assessed using confocal microscope (Zeiss, Germany).

### Preparation of animal model

The animal model for subcutaneous melanoma was established using male C57BL/6 mice aging 6 weeks. B16F10 cells, at a concentration of approximately 10^7^ cells per mouse, were subcutaneously inoculated into the right hind leg root. For the lung metastasis model, B16F10 cells were intravenously injected.

### *In vitro* and* in vivo* imaging

For *in vitro* imaging, 200 µL NDs were transferred into a 1.5 mL centrifuge tube. Therapeutic US was administered, then grayscale US and CEUS images were immediately monitored using a small animal US system (FUJIFILM VisualSonics Vevo3100, Canada). We selected the US parameters (3 W/cm^2^, 1 MHz, 10% duty cycle, 1 min) with the best ADV/imaging for *in vitro* experiments, including ADV/imaging and SDT. As for *in vivo* imaging, the tumors were located using grayscale US. NDs (200 µL) were injected into each mouse via the tail vein, and therapeutic US was applied after 40 min. Immediately after therapeutic US radiation, real-time dynamic changes in tumors were detected by grayscale US and CEUS. To monitor the drug delivery, LA@P-Ce6 NDs were injected through tail veins (200 μL). Real-time dynamic changes in tumors were continuously detected post injection by PA imaging using FUJIFILM VisualSonics Vevo3100. To monitor biodistribution and metabolism, LA@P-Ce6 NDs were injected through tail vein (200 μL). After drug injection, mice were put euthanasia at different time points, their organs were collected for fluorescence imaging using an IVIS Lumina III.

### *In vivo* therapy

All tumor-bearing mice were randomly divided into four groups as Control group, LA@P NDs + US group, Ce6 + US group, and LA@P-Ce6 NDs + US group. The injection volume was calculated based on mouse body weight to achieve a Ce6 dose of 2 mg/kg. Following the administration of the respective drugs or PBS via tail vein injection, therapeutic US (3 W/cm^2^, 1 MHz, 10% duty cycle, 2 min) was applied to the tumor area 40 min after drug injection. These treatments were conducted every 3 days for a total of 3 sessions. Prior to each treatment, the mice were weighed, and the tumor sizes were recorded. After treatments, tumors were harvested for different stainings to study anticancer efficiency *in vivo*.

### Immunological assessment

Tumor tissues were harvested for immunofluorescence staining to study the expression of immune-related markers. Tumor-draining lymph nodes and spleens were collected, and single-cell suspensions were prepared for flow cytometry analysis of immune cells. In addition, RNA sequencing was conducted to tumor tissues to evaluate transcriptomic changes and immune-related gene expression profiles.

### Biosafety assessment

Hemolysis assay was performed to evaluate the blood compatibility of both types of the NDs. Major organs, including the heart, liver, spleen, lung, and kidney, were collected for histological examination by hematoxylin and eosin (H&E) staining.

### Statistical analysis

The software GraphPad 9 (GraphPad, La Jolla, CA, USA) was used for all statistical analysis and visualization. Statistical differences were evaluated using the Student's t-test or a one-way ANOVA according to data types. All data are presented as the mean with SD from at least 3 individual experiments. Significant difference was taken into account when *p < 0.05, **p < 0.01, ***p < 0.001 and ****p < 0.0001.

## Results and Discussion

### Preparation and characterization of LA@P NDs and LA@P-Ce6 NDs

**Figure 2:**
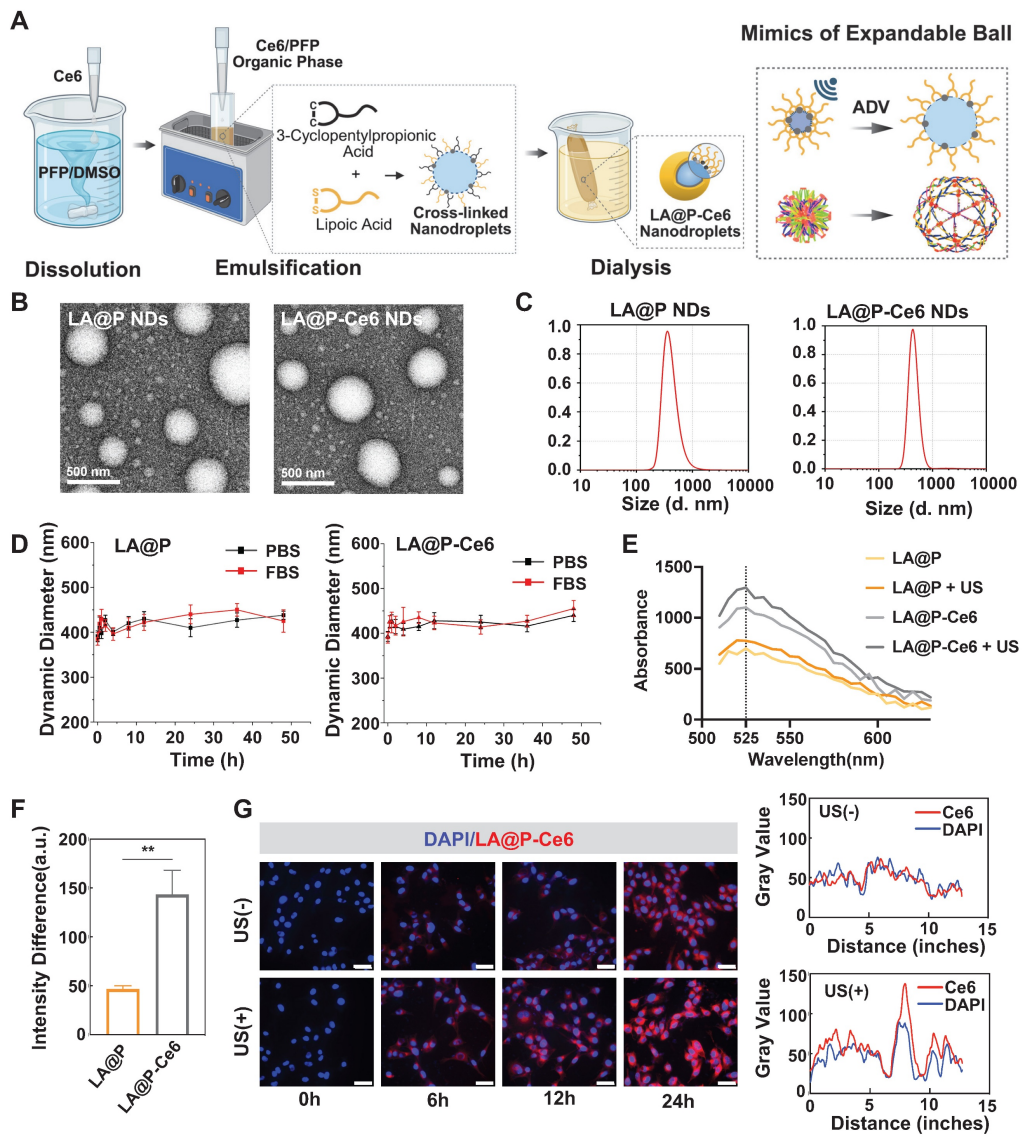
Synthesis and characteristics of LA@P and LA@P-Ce6 NDs. (A) Schematic illustration of the preparation of LA@P-Ce6 NDs. (B) TEM images of LA@P NDs and LA@P-Ce6 NDs. (Scale bar = 500 nm) (C) Size distribution of LA@P NDs and LA@P-Ce6 NDs. (D) Size change of LA@P NDs and LA@P-Ce6 NDs incubated in PBS and FBS in 48 h. (E) ^1^O_2_ generation under US irradiation detected using SOSG reagent. (F) Quantitative fluorescence analysis of ^1^O_2_ production by LA@P NDs and LA@P-Ce6 NDs with/without US irradiation. (G) Co-localization of nuclei/LA@P-Ce6 NDs, and co-localization analysis of 24h groups. (DAPI/Ce6, blue/red; scale bar = 50 μm) Data are presented as mean ± SD (n = 3). ns: not significant, *p < 0.05, **p < 0.01, ***p < 0.001, and ****p < 0.0001.

Most studies using LA as DDSs have only focused on the stability of the LA-based carriers 47. As is shown in Figure 2A, our study advanced the synthesis process and endowed the LA-based drug carrier with elasticity by mimicking the expand-and-contract mechanism of expandable balls. This attempt not only enabled effective drug encapsulation, but also facilitated the ADV of PFP. To prepare elastic shells, we used 3-cyclopentylpropionic acid, which bears structural similarity to LA but lacks the ability to form S-S bonds, as a placeholder during the synthesis process. The interspersed distribution of LA and 3-cyclopentylpropionic acid lengthened the connections between LA molecules and forbade them from creating a tightly packed structure. Therefore, elastic bonds were formed among LA molecules. After dialysis to remove 3-cyclopentylpropionic acid, the resulting LA shell exhibited elasticity and expandability. This elasticity is expected to allow the NDs to perform ADV under mild US irradiation.

Synthesizing stable and appropriately sized NDs was essential for achieving theranostic utilization. These NDs, created an opaque liquid using a modified water-in-oil method, were milky white before drug-loading and turned light gray after loading Ce6, indicating the successful encapsulation of Ce6 into the NDs (Figure S1). The UV-Vis spectrum of LA@P-Ce6 NDs showed a combined pattern of Ce6 and LA@P NDs, evidencing their co-loading (Figure S2). TEM images indicated that both LA@P NDs and LA@P-Ce6 NDs were uniformly dispersed and distributed in spherical shapes (Figure 2B). Figure 2C showed that the particle size of LA@P NDs was around 365 nm, and the particle size of LA@P-Ce6 NDs was around 412 nm. Both NDs remained within the size range for effective permeation through the tumor's vascular endothelium by EPR effect and accumulation at the tumor site 48. Stability tests in PBS and FBS (10%) showed minimal changes in particle sizes over 48h (Figure 2D). The consistent negative charge of the NDs (Figure S3) prevented aggregation and allowed for extended retention times, which therefore could improve therapeutic efficacy within tissues 49.

We synthesized elastic NDs in order to realize ADV under mild US irradiation for imaging 50. Microscopic observations post-US irradiation revealed significant increases in the sizes of LA@P NDs and LA@P-Ce6 NDs, indicating a successful ADV (Figure S4). Comparing to rigid shells, our elastic LA shells guaranteed effective expansion of the NDs under mild US irradiation by lowering the mechanical constraint of the shells, which established the foundation for their imaging ability.

Since US-induced ROS generation is the core of SDT 51, we assessed whether the incorporated Ce6 maintained its sonosensitizing ability within the LA@P-Ce6 NDs. The ^1^O_2_ generation of Ce6-loaded and unloaded NDs before and after US irradiation was quantified using the SOSG fluorescence assay. As is shown in Figure 2E, LA@P-Ce6 NDs exhibited the highest fluorescence emission at 525 nm under US irradiation, and the fluorescence intensity difference before and after US exposure of LA@P-Ce6 NDs was significantly greater than that of LA@P NDs (Figure 2F), confirming their superior ROS-generating capability. This further confirmed that Ce6 was successfully loaded into the LA@P-Ce6 NDs and kept the function as a sonosensitizer. In LA@P-Ce6, the concentration of Ce6 was 160 µg/mL, and the concentration of LA was 41.8 mg/mL. This effective Ce6 load ensured efficient ^1^O_2_ generation, thereby highlighting the potential of LA@P-Ce6 NDs to display SDT effects in therapeutic applications.

Effective intracellular uptake was critical for ensuring that our NDs can deliver drugs into cells to realize the therapeutic effects. Although NDs were primarily internalized by cells via passive diffusion, the US-induced vaporization of PFP transformed them into MBs and enabled them to exploit the UTMD effect, during which they could increase membrane permeability through cavitation and sonoporation 52. Figure 2G revealed a progressive increase in the intracellular fluorescence signal over time, indicating successful internalization of the drugs. Cells received therapeutic US irradiation showed markedly stronger red fluorescence, and corresponding co-localization analysis confirmed that US-irradiated group exhibited relatively higher fluorescence intensity comparing to non-irradiated group after 24h. These findings suggested that US facilitated cellular uptake of LA@P-Ce6 NDs, which may enhance intracellular drug delivery and potentially lead to more effective anti-melanoma therapy.

### *In vitro* antitumor effect of LA@P-Ce6 NDs

**Figure 3:**
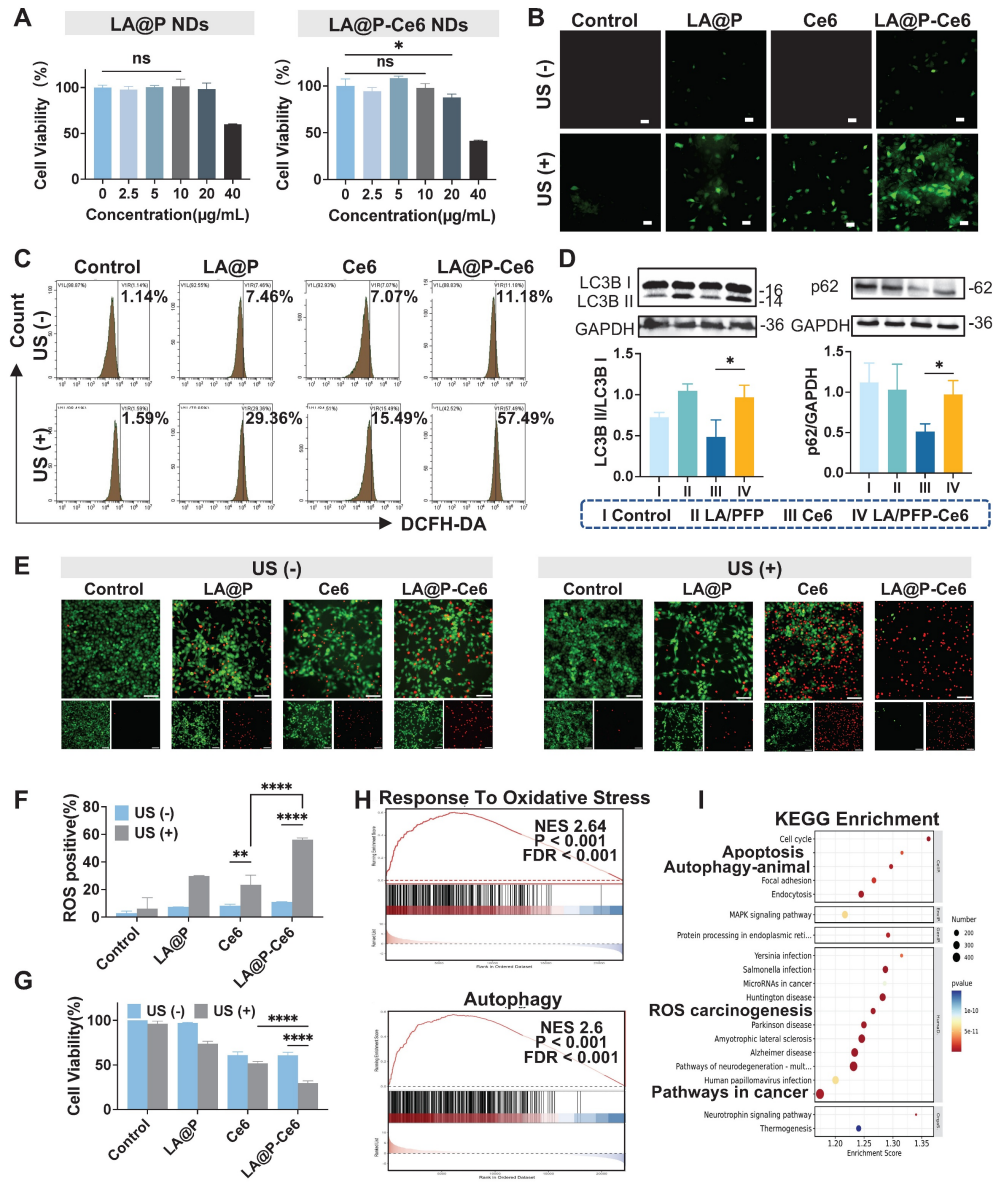
*In vitro* evaluation of the therapeutic mechanism and effecacy of LA@P-Ce6 NDs. (A) Cell viability of HUVECs after incubation with various concentrations of LA@P NDs andLA@P-Ce6 NDs for 48 h, determined by CCK-8 assay. (B) Fluorescence microscopy images of intracellular ROS generation in B16F10 cells under different treatments, detected using DCFH-DA probe. (Scale bar = 50 μm) (C) Flow cytometry analysis of ROS levels in B16F10 cells under different treatments. (D) Western blot analysis of LC3B and p62 expression in B16F10 cells under different treatments. (E) Live/dead cell staining images of B16F10 cells under different treatments. (Calcein-AM/PI, green/red; scale bar = 100 μm) (F) Corresponding statistical analysis of ROS positive cells from flow cytometry analysis. (G) Cell viability of B16F10 cells under different treatments determined by CCK-8 assay. (H) GSEA of oxidative stress- and autophagy-related pathways. (I) KEGG enrichment analysis showing the top 20 significantly enriched pathways related to the treatment. Data are presented as mean ± SD (n = 3). ns: not significant, *p < 0.05, **p < 0.01, ****p < 0.0001.

Ensuring the biocompatibility and biosafety is the prerequisite for therapeutic applications. Since previous studies indicated that both LA and Ce6 exhibited good biocompatibility 53-55, we examined the biosafety of the synthesized LA@P NDs and LA@P-Ce6 NDs. As is shown in Figure 3A, *in vitro* CCK-8 assays showed that at the concentrations of LA equal to or below 10 µg/mL, the cell viability of HUVECs remained over 90% after 48 h of exposure to either LA@P NDs or LA@P-Ce6 NDs, indicating minimal cytotoxicity at this therapeutic concentration.

Regulating the ROS level in tumor tissues has emerged as a promising therapeutic strategy for cancer treatment 56, 57. Interestingly, many studies have proposed that Ce6 is an efficient organic sonosensitizer, while LA also has the capacity to generate ROS in TME 53. Herein, the DCFH-DA probe was administered to detect the intracellular ROS level and further explore whether there is a synergistic effect in ROS production between LA and Ce6 58. As is shown in Figure 3B, both LA@P NDs group and LA@P NDs + US group showed moderate fluorescence intensity. Ce6 + US group showed bright fluorescence, and LA@P-Ce6 NDs + US group demonstrated the strongest fluorescence signal, indicating the most significant ROS generation. This trend was also observed in flow cytometry analysis in Figure 3C and F, where the LA@P-Ce6 NDs group exhibited significantly more ROS positive cells.

In the TME, autophagy is closely linked to ROS levels 59, 60. While high ROS level can promote cell death, it can also stimulate cytoprotective autophagy, which may facilitate tumor progression and induce therapy resistance 39, 61. Previous studies have reported that LA treatment can interfere with autophagic flux in several cell lines by impairing autophagic flux at the late stage 42, 62, 63. To investigate whether LA exhibited similar treating mechanism, we examined LC3B and p62 expression in different treatment groups. As is shown in Figure 3D, significant accumulation of LC3B-II and p62 was detected in the LA@P-Ce6 NDs group comparing to Ce6 group, indicating impaired autophagic flux. To visualize the autophagy status, we introduced the tandem mCherry-EGFP-LC3 reporter into B16F10 cells. As is shown in Figure S5, Ce6 + US treatment group exhibited mostly red puncta, while LA@P-Ce6 NDs group exhibited more yellow puncta. The accumulation of LC3B and p62, together with the predominance of yellow puncta, indicated that LA blocked autophagic flux at a late stage during SDT.

We next evaluated whether the enhanced ROS production and autophagy modulation translated into increased tumor cell death. Live/dead cell staining (Figure 3E) revealed that the LA@P NDs and LA@P NDs + US groups showed minimal cell death, with only sparse red signals observed. Ce6 + US treatment induced a moderate proportion of dead cells, whereas LA@P-Ce6 NDs + US treatment led to extensive cell death, as indicated by the predominance of red fluorescence. Similarly, after incubation with CCK-8 reagent for 2 h, the cell viability of the LA@P-Ce6 + US group was calculated to be significantly lower than Ce6 + US group (Figure 3G). These results demonstrated that LA@P-Ce6 NDs could effectively integrate SDT and autophagy modulation to achieve enhanced melanoma cell killing.

To further study the molecular basis of the observed mechanisms, we compared the transcriptomic profiles of LA@P-Ce6 + US group with untreated group. The heat map (Figure S6) showed a broad spectrum of differentially expressed genes (DEGs). Gene set enrichment analysis (GSEA) revealed significant enrichment in reaction to oxidative stress and autophagy pathways (Figure 3H), consistent with the ROS generation and autophagy modulation observed experimentally. A Sankey plot further illustrated the interconnections among oxidative stress-related and autophagy-related genes (Figure S7). In addition, KEGG pathway enrichment identified apoptosis, autophagy, chemical carcinogenesis and cancer-related pathways to be within the top 20 enriched categories (Figure 3I). Collectively, these transcriptomic findings reinforce the mechanistic basis of ROS production, autophagy modulation, and subsequent cell death triggered by LA@P-Ce6 NDs + US treatment.

### *In vitro* immune activation of LA@P-Ce6 NDs

**Figure 4:**
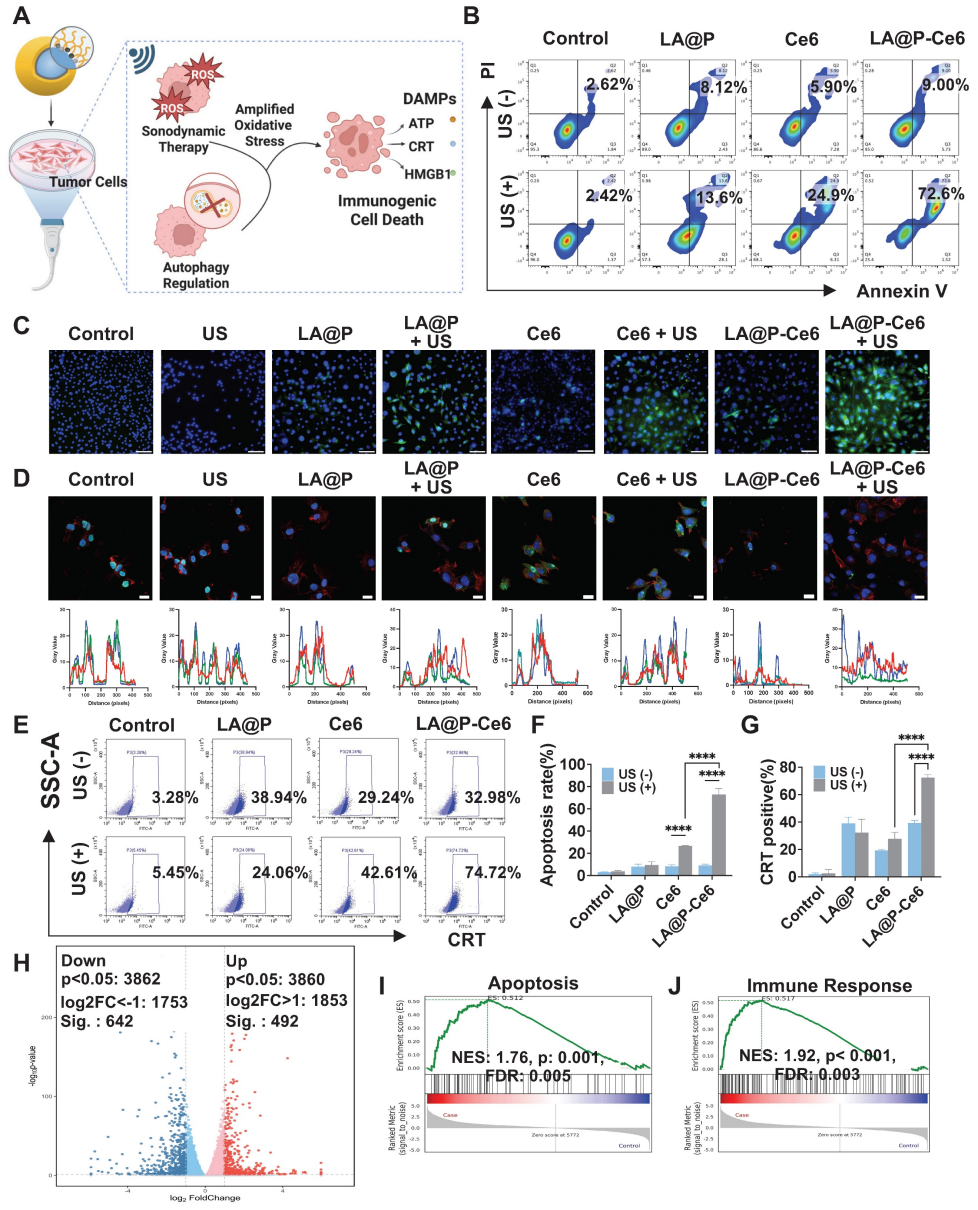
*In vitro* evaluation of immune activation. (A) Schematic illustration of LA@P-Ce6 NDs mediated ICD. (B) Flow cytometry analysis of FITC/PI channels of B16F10 cells under different treatments. (C) Immunofluorescence staining images showing CRT expression of B16F10 cells under different treatments. (CRT/DAPI, green/blue; scale bar = 100 μm). (D) CLSM images showing HMGB1 release and co-localization analysis. (HMGB1/phalloidin/DAPI, green/red/blue; scale bar = 20 μm) (E) Flow cytometry analysis of CRT expression of B16F10 cells under different treatments. (F) Corresponding statistical analysis of cell apoptosis from flow cytometry analysis. (G) Corresponding statistical analysis of CRT expression from flow cytometry analysis. (H) Volcano plot showing differential gene expression between treatment group and control group. (I) GSEA of apoptosis-related pathways. (J) GSEA of immune response-related pathways. Data are presented as mean ± SD (n = 3). ns: not significant, ****p < 0.0001.

Based on the findings that the synergy of autophagy modulation and SDT induced amplified oxidative stress and subsequently increased cytotoxicity, we further investigated the ICD induction (Figure 4A). We assessed cell apoptosis by flow cytometry using Annexin-V/PI staining (Figure 4B). The results revealed that both Ce6 and LA@P-Ce6 treated cells exhibited increased Annexin-V/PI positive cells under US irradiation comparing to their respective non-irradiated groups. Statistical analysis (Figure 4F) confirmed that LA@P-Ce6 NDs + US treatment induced significantly more Annexin-V/PI positive cells than Ce6 + US treatment, consistent with the stronger ROS generation.

A critical determinant of whether cell death is immunogenic is the emission of DAMPs 61. We therefore examined ICD-related DAMPs, including CRT exposure, HMGB1 release, and extracellular ATP level 64. As is shown in immunofluorescence staining results (Figure 4C) and flow cytometry analyses (Figure 4E, G), LA@P-Ce6 NDs + US treatment induced the most pronounced surface CRT exposure, whereas control and LA@P NDs groups exhibited negligible signals. Confocal imaging (Figure 4D) further showed significant nuclear-to-cytoplasmic translocation and extracellular release of HMGB1 in LA@P-Ce6 NDs + US group, while other groups showed less translocation. Moreover, supernatant ATP concentration analysis (Figure S8) revealed elevated extracellular ATP level in LA@P-Ce6 NDs + US group, significantly higher than that of Ce6 + US group. These results demonstrated that LA@P-Ce6 NDs + US not only induced cell death but also triggered ICD.

To gain further insight into the cellular and immunological consequences of our treatment, we looked into the RNA sequencing (RNA-seq) analysis results of LA@P-Ce6 NDs + US group comparing to control group. The volcano analysis revealed abundant DEGs, including upregulated genes and down regulated genes (Figure 4H). GO enrichment analysis revealed marked alterations in pathways related to oxidative stress, autophagy, and apoptosis (Figure S9), as well as their interrelationships with the regulation of immune response (Figure S10). GSEA (Figure 4I, J) revealed significant enrichment in pathways associated with apoptosis and immune responses, consistent with the previous experimental results. Specifically, GSEA of immune-associated pathways such as natural killer (NK) cell mediated cytotoxicity and T cell differentiation (Figure S11) also implied that the treatment enhanced cytotoxicity and initiated immune response in RNA level. These results suggested that our therapy not only caused tumor cell death but also boosted anti-tumor immunity, providing a cellular-level foundation for the subsequent *in vivo* studies on primary and metastatic melanoma inhibition.

### Imaging performances of the NDs

**Figure 5:**
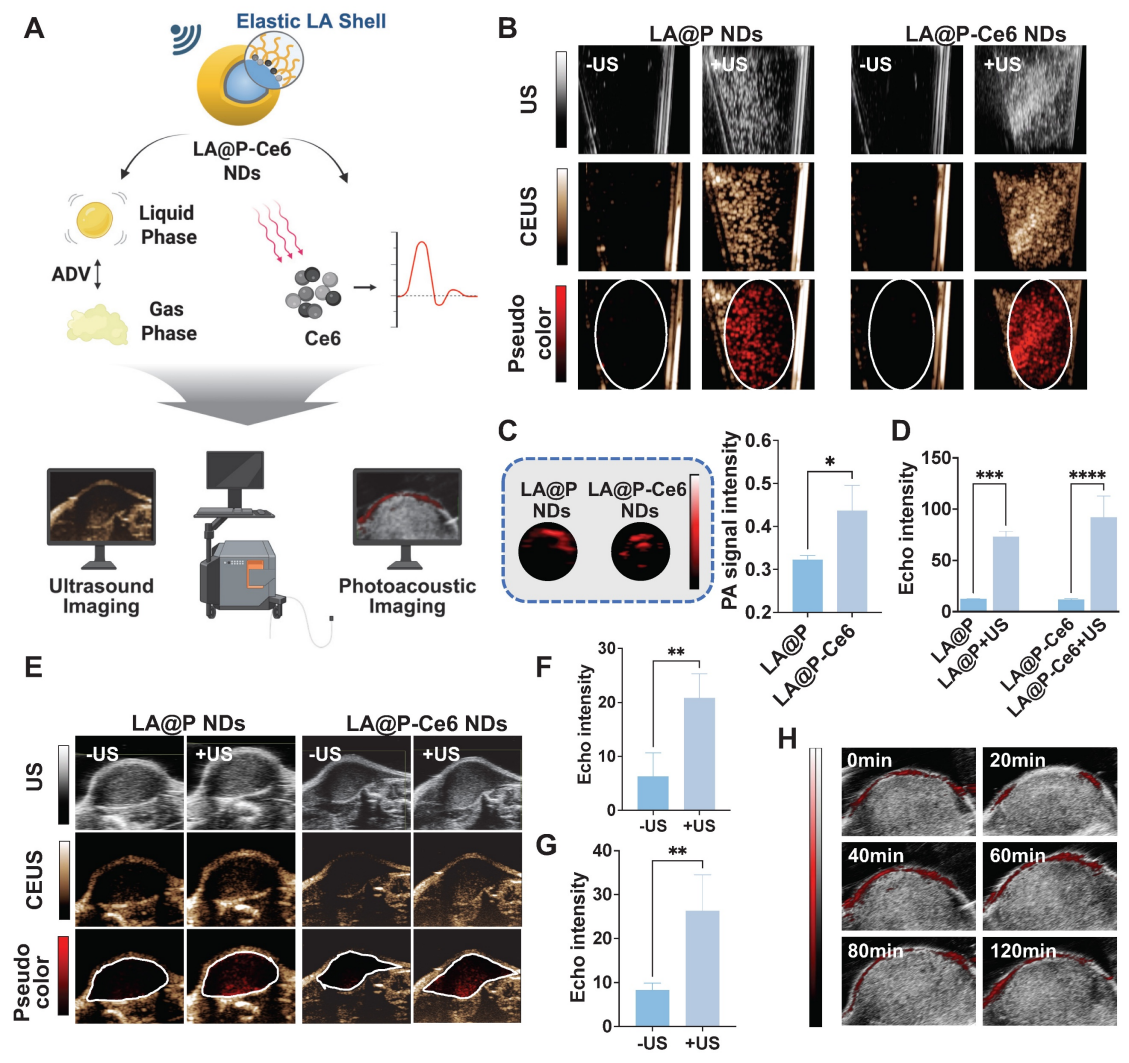
*In vitro* and *in vivo* CEUS and PA imaging performances. (A) Schematic illustration of the CEUS/PA imaging mechanism of LA@P-Ce6 NDs. (B) *In vitro* US and CEUS imaging of LA@P NDs and LA@P-Ce6 NDs, with corresponding pseudo-color CEUS images. (C) *In vitro* PA imaging of LA@P NDs and LA@P-Ce6 NDs, and corresponding quantitative analysis of PA signal intensity. (D) Corresponding quantitative analysis of *in vitro* CEUS signal intensity. (E) *In vivo* US, CEUS, and pseudo-color CEUS imaging of subcutaneous B16F10 tumors. (F-G) Corresponding quantitative analysis of *in vivo* CEUS signal intensity of (F) LA@P NDs and (G) LA@P-Ce6 NDs. (H) *In vivo* PA imaging of subcutaneous tumors. Data are presented as mean ± SD (n = 3). *p < 0.05, **p < 0.01, ***p < 0.001, ****p < 0.0001.

Given that the imaging performance of MBs is closely related to the properties of both core and shell 26, we designed NDs with a liquid PFP core and an elastic LA shell. PFP, among all types of liquid PFC, is frequently selected as the phase-change agent due to its low boiling point, which allows for efficient ADV under relatively low acoustic intensities 65, thereby minimizing potential tissue damage while enabling effective imaging. PFP is the core for US imaging in NDs by performing ADV. The elastic nature of the LA shell provided sufficient stability for the NDs in their liquid form, where they generated negligible background signals, while facilitating efficient ADV to generate echogenic MBs under US irradiation (Figure 5A). *In vitro*, as is shown in Figure 5B, negligible signals were observed before US exposure, whereas both LA@P NDs and LA@P-Ce6 NDs produced noticeable signals post-irradiation in US imaging, and CEUS mode provided more pronounced visualization of these signals. Quantitative analysis of CEUS (Figure 5D) revealed that the echo intensities increased significantly after US exposure for both types of NDs, which could be attributed to the elasticity of their shells to allow PFP to perform ADV. This result was consistent with the ADV-driven MB formation and size expansion observed previously. Furthermore, since Ce6 is acknowledged to possess intrinsic PA properties 66, we evaluated the PA signal of LA@P-Ce6 NDs *in vitro* (Figure 5A). As expected, LA@P-Ce6 NDs exhibited strong PA signals compared with LA@P NDs (Figure 5C and S12), further confirming their suitability as US/PA contrast agents. These results collectively established a solid basis for subsequent i*n vivo* imaging studies.

Then we used subcutaneous tumors for *in vivo* imaging. Following intravenous injection, the NDs were expected to circulate systemically and accumulate within tumor tissues via the EPR effect. Considering that the high melanin content in melanoma lesions may interfere with fluorescence imaging 67, PA imaging was employed to monitor the accumulation of NDs in subcutaneous tumors. We monitored the relative change in PA signals over time to determine the treatment time window to apply therapeutic US.

As is shown in Figure 5H and S13, low PA signal was detected in tumors in 20 min post-injection, while a marked signal appeared at 40 min post-injection, confirming the effective accumulation of NDs within the tumor site. The signal intensity began to decrease 60 min post-injection and beyond, suggesting partial clearance and providing a time reference for US-triggered phase transition. Based on this finding, therapeutic US was scheduled to administer in 40 min post-injection to explore the imaging effect of the NDs as US contrast agents in subcutaneous tumors. As is shown in Figure 5E, both LA@P NDs and LA@P-Ce6 NDs produced remarkable CEUS enhancement upon US irradiation, whereas the baseline signals before US were minimal but not zero, likely due to intrinsic tumor tissue components or that a small portion of NDs vaporized under physiological temperature. At 80 min post-injection, CEUS signals of both types of NDs were reduced, indicating that the NDs might have gone through disruption and drug release in TME (Figure S14). Quantitative analysis confirmed that the CEUS signal intensities of both types of NDs increased significantly following US irradiation (Figure 5F, G). These *in vivo* findings demonstrated that PA monitoring successfully identified the temporal accumulation profile of the NDs for US activated CEUS imaging. Together, these results underscored the theranostic potential of LA@P-Ce6 NDs as a multifunctional platform integrating drug delivery, multimodal imaging, and US-triggered therapy.

### *In vivo* anti-cancer evaluation of LA@P-Ce6 NDs

**Figure 6:**
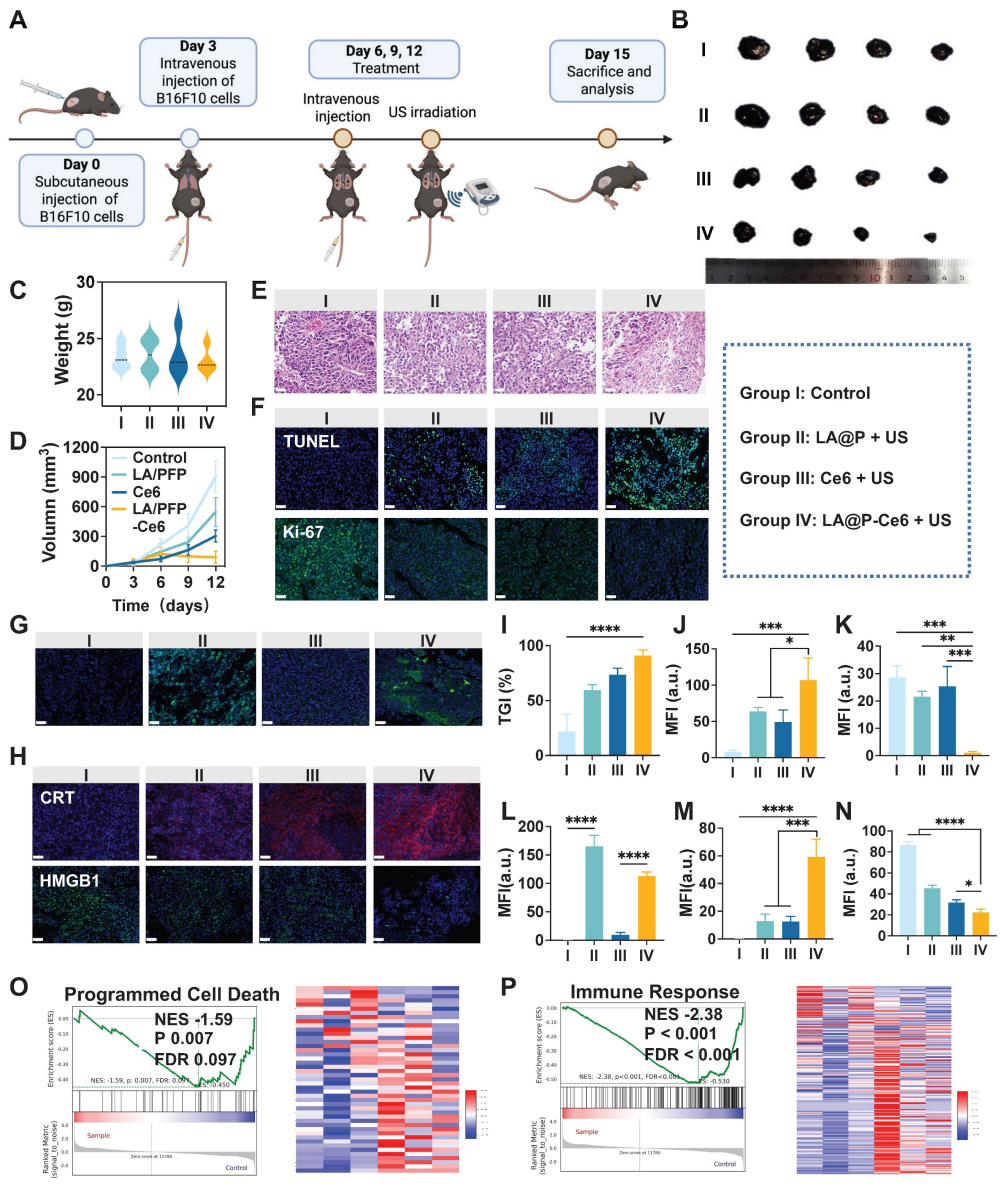
*In vivo* antitumor efficacy of LA@P-Ce6 NDs. (A) Schematic illustration of the treatment schedule. (B) Photographs of the tumors in different treatment groups. (C) Body weights of mice at the end of the treatments. (n = 4) (D) Tumor volume growth curves of different treatment groups. (n = 4) (E) H&E staining of the tumor tissues at the end of the study in different treatment groups. (Scale bar = 40 μm) (F) Immunofluorescence staining of tumor tissues of TUNEL and Ki-67 to assess apoptosis and proliferation. (Scale bar = 50 μm) (G) Immunofluorescence staining of LC3B to evaluate autophagy levels in tumor tissues. (LC3B, green; scale bar = 50 μm) (H) Immunofluorescence staining of DAMPs, including CRT and HMGB1. (Scale bar = 50 μm) (I) Tumor inhibition rates in different treatment groups. (J-N) Quantification of mean fluorescence intensity (MFI) corresponding to the immunofluorescence images in F, H, and I. (n = 3) (P) GSEA of programmed cell death-related pathways. (Q) GSEA of immune response-related gene sets. Group I: Control; Group II: LA@P NDs + US; Group III: Ce6 + US; Group IV: LA@P-Ce6 NDs + US. Data are presented as mean ± SD. *p < 0.05, **p < 0.01, ***p < 0.001, ****p < 0.0001.

Based on the encouraging results from *in vitro* experiments and multimodal imaging, we next evaluated the therapeutic performances of LA@P-Ce6 NDs in murine subcutaneous melanoma models. As is shown in Figure 6A, mice received intravenous injections every three days for a total of three cycles, with therapeutic US administered 40 min post-injection. Tumor volumes were measured prior to each treatment cycle, and tumors were harvested at the end of the study for further analyses. As is shown in Figure 6B, tumors from the control group were the largest, while those from the LA@P NDs group and the Ce6 group were moderately reduced. LA@P-Ce6 NDs group exhibited the smallest tumor masses, suggesting the strongest therapeutic effect. The tumor growth curve (Figure 6D) and the tumor inhibition rates (Figure 6I) further confirmed the superior antitumor efficacy of the LA@P-Ce6 NDs treatment. Histological evaluation by H&E staining (Figure 6E) revealed that tumors in the control group exhibited dense cell arrangement and abundant blood supply, indicating vigorous tumor proliferation. In contrast, tumors in the LA@P-Ce6 NDs group displayed damaged cell morphology and sparse cell distribution. Consistent with these findings, TUNEL staining demonstrated markedly higher apoptotic signals in the LA@P-Ce6 NDs group comparing to other groups (Figure 6F). Moreover, Ki-67 staining showed the highest proliferative activity in the control group, moderate reduction in the LA@P NDs and Ce6 groups, and the lowest expression in the LA@P-Ce6 NDs group (Figure 6F). Quantitative analysis (Figure 6J, K) further validated that LA@P-Ce6 NDs under US irradiation induced the strongest apoptosis and suppression of proliferation. Meanwhile, subsequent immunofluorescence staining of LC3B confirmed the modulation of autophagy in LA@P-Ce6 NDs group, in accordance with *in vitro* autophagy study (Figure 6G, L).

We next examined whether ICD could be observed *in vivo*. Immunofluorescence staining revealed that the LA@P-Ce6 NDs group exhibited the strongest CRT exposure on the cell surface (Figure 6H, M) and HMGB1 reduction in cells (Figure 6H, N), both of which are hallmarks of ICD. These results confirm that the therapeutic efficacy of LA@P-Ce6 NDs was accompanied by the induction of ICD *in vivo*, thereby linking cytotoxicity with the activation of antitumor immunity. Transcriptomic profiling of tumor tissues revealed significant enrichment in apoptotic process from LA@P-Ce6 NDs group (Figure S15). Furthermore, GSEA demonstrated strong enrichment of gene sets associated with programmed cell death (Figure 6O) and immune response (Figure 6P), further supporting that LA@P-Ce6 NDs not only induced direct cytotoxicity but also potentiated immunogenic pathways.

To evaluate the biosafety of our treatment, we first assessed hemolytic activity of the drugs, and all groups exhibited hemolysis rates below 5%, ensuring their safety for intravenous injection (Figure S16). No significant differences in body weights among four groups were observed (Figure 6C, S17), which further supported the biosafety of the treatments. Additional analyses, including serum biochemical tests of liver and kidney function (Figure S18) as well as H&E staining (Figure S19) showed no appreciable pathological alterations. Biodistribution studies revealed that fluorescence signals mainly accumulated in the liver and lung shortly after injection, but became nearly undetectable at 24 h (Figure S20), indicating effective clearance of the NDs from the body. Collectively, these results demonstrated that LA@P-Ce6 NDs are safe and metabolizable, thus suitable for systemic administration and repeated therapeutic use.

### *In vivo* immune activation of LA@P-Ce6 NDs

**Figure 7:**
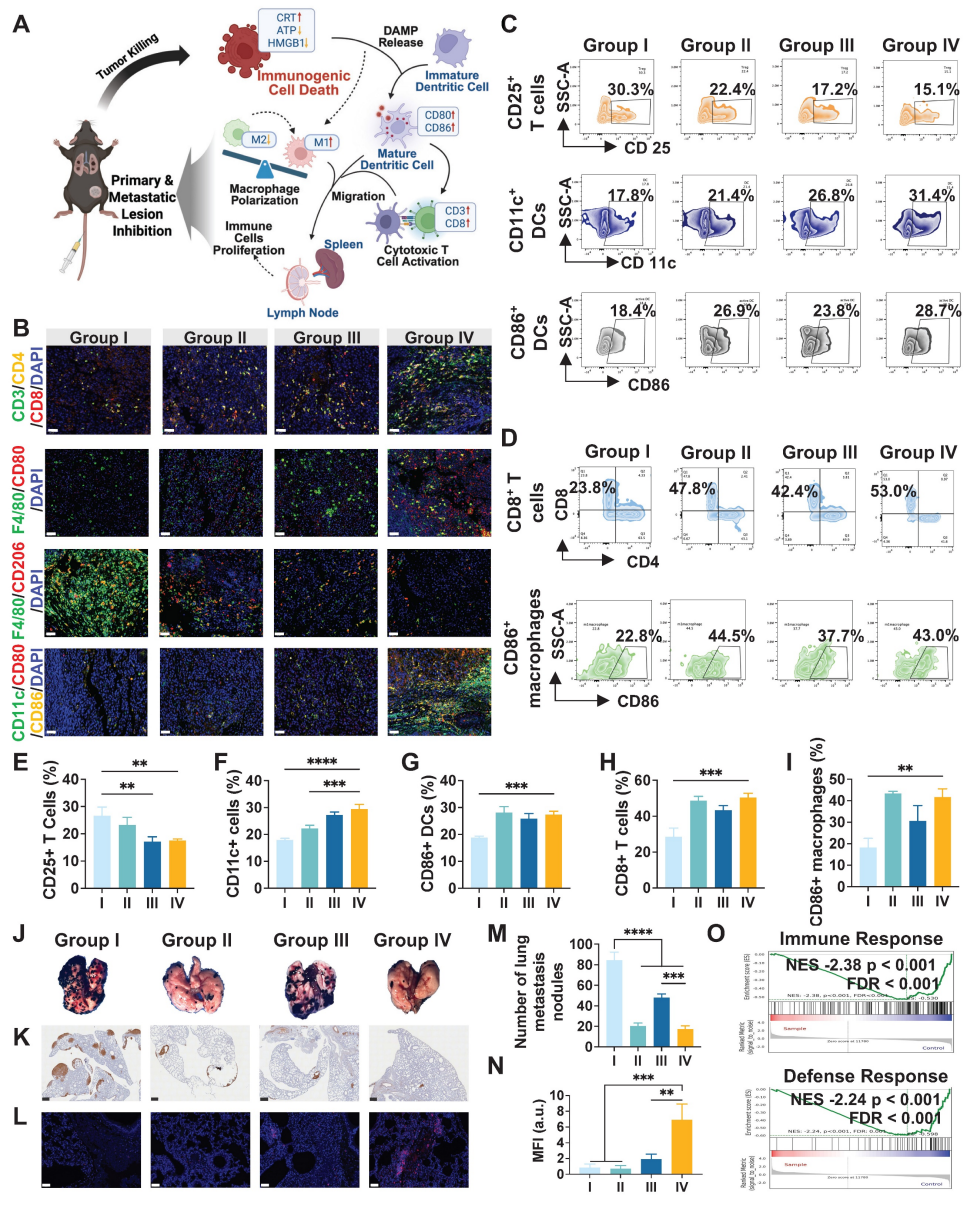
*In vivo* evaluation of immune activation and anti-metastatic efficacy. (A) Schematic illustration of immune activation. (B) Multi-immunofluorescence staining of subcutaneous tumor tissues including CD3, CD4, and CD8 staining, F4/80 and CD80 staining, F4/80 and CD206 staining, and CD11c, CD80, and CD86 staining. (Scale bar = 50 μm) (C) Flow cytometry analysis of immune cell populations in spleens, including CD25^+^ T cells, CD11c^+^ DCs, and CD86^+^ DCs. (D) Flow cytometry analysis of immune cells in draining lymph nodes, including CD8^+^ T cells and CD86^+^ macrophages. (E-I) Corresponding quantitative statistical analysis of flow cytometry analysis in C and D. (J) Representative images of lungs after treatment. (K) S100 immunohistochemical staining of lung tissues. (Scale bar = 500 μm) (L) Immunofluorescence staining of CD8^+^ T cells in lung tissues. (Scale bar = 50 μm) (M) Quantitative analysis of metastatic lung nodules. (N) MFI analysis of CD8^+^ T cell staining. (O) GSEA of immune response- and defense response-related gene sets. Group I: Control; Group II: LA@P NDs + US; Group III: Ce6 + US; Group IV: LA@P-Ce6 NDs + US. Data are presented as mean ± SD (n = 3). **p < 0.01, ***p < 0.001, ****p < 0.0001.

Building on the observation that the therapy induced ICD in subcutaneous tumors, we sought to determine whether this therapy could enhance anti-tumor immunity *in vivo* and suppress lung metastasis (Figure 7A). As is shown in Figure 7B, multiplex immunofluorescence staining of tumor tissues revealed that the LA@P-Ce6 NDs group exhibited the highest infiltration of CD8^+^ T cells and CD80^+^ macrophages, along with increased CD80^+^CD86^+^ dendritic cells (DCs), whereas the proportion of CD206^+^ macrophages was markedly reduced. Flow cytometry analysis of spleens demonstrated that the treatment of LA@P-Ce6 NDs + US significantly increased the percentage of CD25^+^ T cells and CD86^+^ DCs, indicating systemic T cell activation and DC maturation, and CD11c^+^ cells also showed a consistent upward trend (Figure 7C, E-G). Analysis of the draining lymph nodes further showed that CD8^+^ T cells and CD86^+^ macrophages were elevated in the LA@P-Ce6 NDs + US group, suggesting that local lymphoid activation contributed to anti-tumor immunity (Figure 7D, H-I). Collectively, these results indicated that LA@P-Ce6 NDs + US therapy not only promoted tumor cell death via ICD but also orchestrated a profound immune response, activating DCs and cytotoxic T cells both locally and systemically.

We evaluated the inhibition of lung metastasis to further study the immune activation of our treatment. Gross lung tissues revealed extensive metastatic nodules in the control and free Ce6 groups, whereas fewer nodules were observed in the LA@P-Ce6 NDs group (Figure 7J). Corresponding statistical analysis of metastatic nodules also proved that LA@P-Ce6 NDs + US treatment achieved the most pronounced suppression of lung metastasis (Figure 7M). Consistently, S100 immunohistochemical staining showed a significant reduction in metastatic melanoma burden in the LA@P-Ce6 NDs + US group compared to other groups (Figure 7K). Immunofluorescence staining of lung tissues further demonstrated enhanced infiltration of CD8^+^ T cells in the LA@P-Ce6 NDs + US group (Figure 7L), which was quantitatively validated by MFI analysis (Figure 7N). GSEA in Figure 7O showed prominent alternations in immune-related pathways, particularly in immune response and defense response. These findings further indicated that our treatment induced strong anti-tumor immune activation, which translated into effective inhibition of lung metastasis.

## Conclusion

In this study, we developed a novel US-responsive DSDS (LA@P-Ce6 NDs) for multimodal image-guided SDT enhanced by autophagy modulation for melanoma management. The elastic shells of the NDs ensured the stable circulation, effective ADV, and on-demand drug release, therefore achieving spatiotemporally controlled therapy. The proper elasticity of the shell laid foundation for US-triggered imaging and therapy. As drug delivery systems with US/PA imaging capabilities, LA@P-Ce6 NDs may enable visualization of subcutaneous melanoma lesions while monitoring drug accumulation and activation during treatment. The synchronized release of LA and activation of Ce6 led to enhanced ROS stress and effective autophagy modulation, therefore realizing synergistic tumor cell killing and ultimately triggering ICD and systemic anti-tumor immunity. Our treatment of subcutaneous melanoma induced significant activation of DCs, T cell priming, and immune-related signaling pathways, molecularly proved to reshaped the TME. The systemic anti-tumor immune effect is further reflected in the significant inhibition of lung metastasis. Collectively, our study established a promising theranostic platform for advanced melanoma. However, our imaging strategies still have limitations. The competitive light absorption between Ce6 and melanin compromises the specificity and quality of NDs' visualization. In our subsequent studies, we aim to optimize imaging strategies to realize more precise image-guided therapy.

## Supplementary Material

Supplementary figures.

## Data Availability

The data that support the findings of this study are available from the corresponding authors upon reasonable request.

## Abbreviations

SDT: sonodynamic therapy
Ce6: chlorin e6
ROS: reactive oxygen species
US: ultrasound
PFP: perfluoropentane
UV: ultraviolet
ADV: acoustic droplet vaporization
PA: photoacoustic
PDT: photodynamic therapy
NIR: near-infrared
ICD: immunogenic cell death
CEUS: contrast-enhanced ultrasound
DAMPs: damage-associated molecular patterns
DDSs: drug delivery systems
MBs: microbubbles
EPR: enhanced permeability and retention
NDs: nanodroplets
PFC: perfluorocarbon
ADV: acoustic droplet vaporization
UTMD: ultrasound-targeted microbubble destruction
DSDSs: drug self-delivery system
TME: tumor microenvironment
LA: lipoic acid
GSH: glutathione
GSEA: gene set enrichment analysis
TEM: transmission electron microscope
RNA-seq: RNA sequencing
DEGs: differentially expressed genes
DC: dendritic cell
PI: propidium Iodide
DCFH-DA: dichlorofluorescein diacetate
HUVEC: human umbilical vein endothelial cell
RBCs: red blood cells
H&E: hematoxylin and eosin
MFI: mean fluorescence intensity
SOSG: singlet oxygen sensor green
