# Supplementary material for: Lipoic acid-engineered nanodroplets for multimodal imaging and enhanced sonodynamic therapy in melanoma treatment
Source: Theranostics. 2026 Mar 9;16(10):5278–95. doi: 10.7150/thno.127554 (PMC13080621; doi:10.7150/thno.127554)
Supplement: Supplementary file 1 — Supplementary figures. [file thnov16p5278s1.pdf]

Supplementary materials

Lipoic acid-engineered nanodroplets for multimodal imaging and enhanced sonodynamic therapy in melanoma treatment

Ziyao Wang[1#], Yongchao Yao[2,3#], Ziyan Feng[1], Yulong Liao[3], Liyun Wang[1], Xueyang Xiao[1], Wenchuang Hu[3], Zhiyong Qian[2], Li Qiu[1]**

[1]Department of Ultrasound, West China Hospital, Sichuan University, Chengdu, 610041, Sichuan, China.

[2]Department of Biotherapy, Cancer Center and State Key Laboratory of Biotherapy, West China Hospital, Sichuan University, Chengdu 610041, Sichuan, China.

[3]Precision Medicine Translational Research Center (PMTRC), West China Hospital, Sichuan University, Chengdu 610041, Sichuan, China.

*Correspondence and requests for materials should be addressed to Zhiyong Qian (email: anderson-qian@163.com) or Li Qiu (email: qiulihx@scu.edu.cn).

[#]Both authors contributed equally to this work.

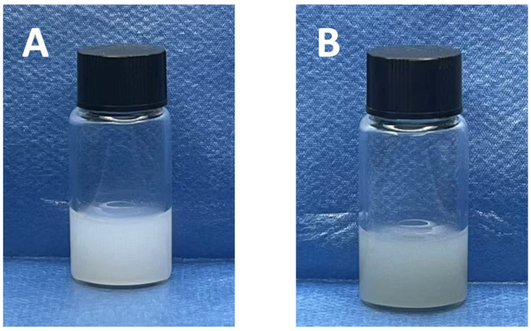

Figure S1. Photographs of (A) LA@P NDs and (B) LA@P-Ce6 NDs.

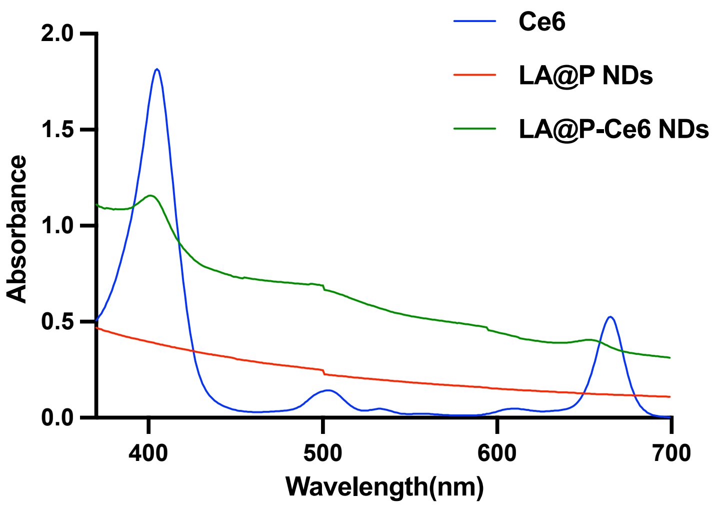

Figure S2. UV-Vis spectrum of Ce6, LA@P NDs and LA@P-Ce6 NDs.

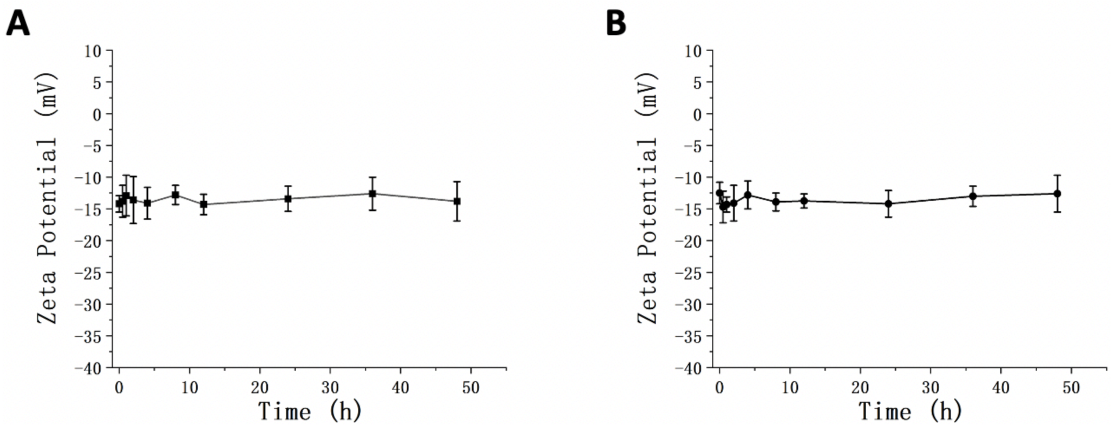

Figure S3. Zeta potential change of (A) LA@P NDs and (B) LA@P-Ce6 NDs in 48 h.

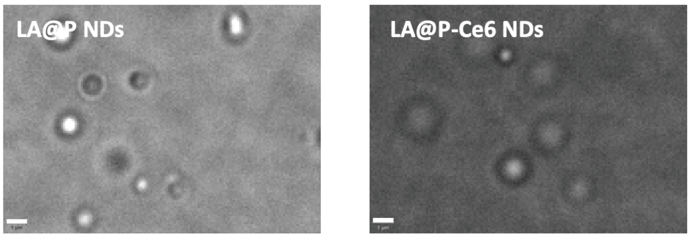

Figure S4. Confocal microscope images of LA@P NDs and LA@P-Ce6 NDs. (Scale bar = 1 μm)

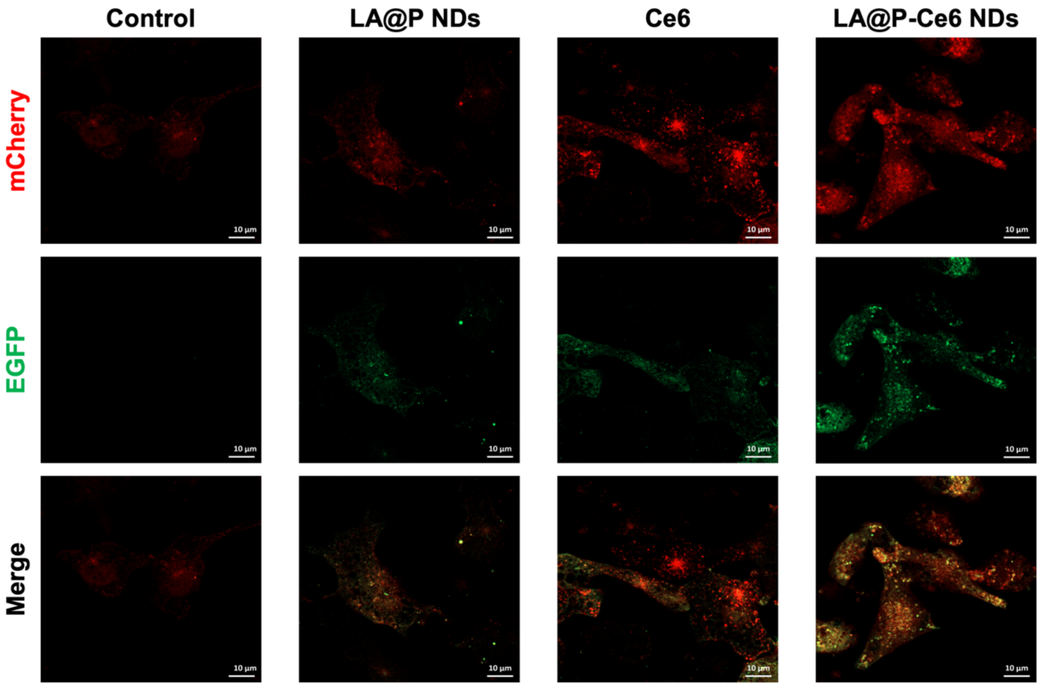

Figure S5. CLSM images of cell autophagic flux monitored by the tandem mCherry-EGFP-LC3 reporter under different treatments. (Red puncta indicating autolysosomes vs. yellow puncta indicating autophagosomes; scale bar=10 μm)

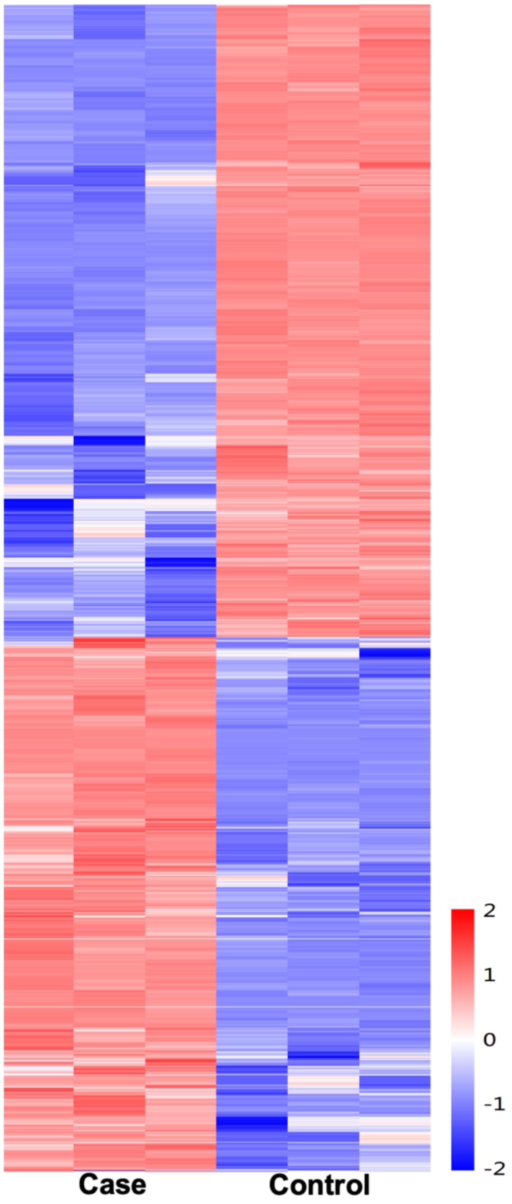

Figure S6. Heatmap of DEGs between LA@P-Ce6 NDs + US group (case group) and Control group.

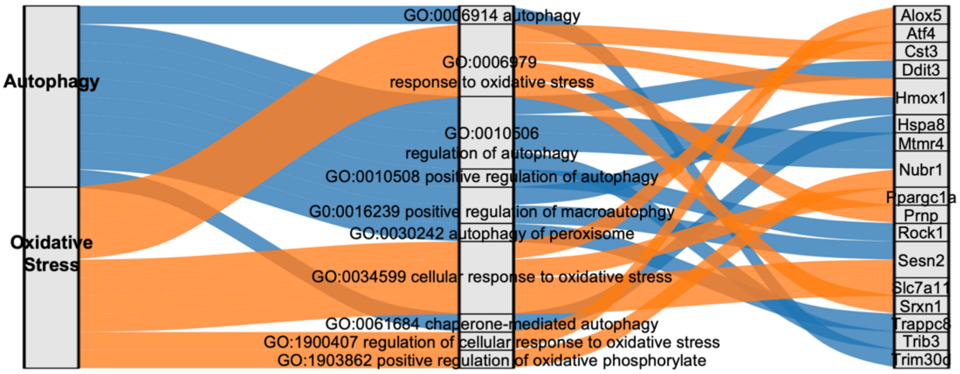

Figure S7. Interconnections between oxidative stress- and autophagy-related pathways at the gene level.

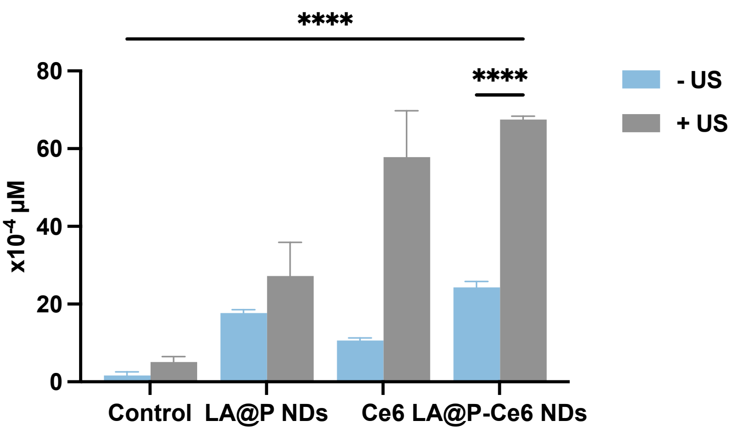

Figure S8. ATP concentration in the supernatant of B16F10 cells under different treatments. Data are presented as mean ± SD. Statistical significance was calculated by one-way ANOVA. ns: not significant, *p < 0.05, **p < 0.01, ***p < 0.001, and ****p < 0.0001.

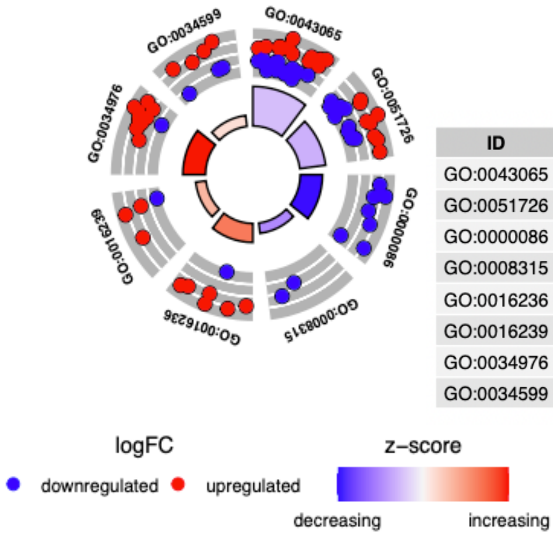

Figure S9. GO enrichment plots of DEGs associated with oxidative stress, autophagy, and apoptosis.

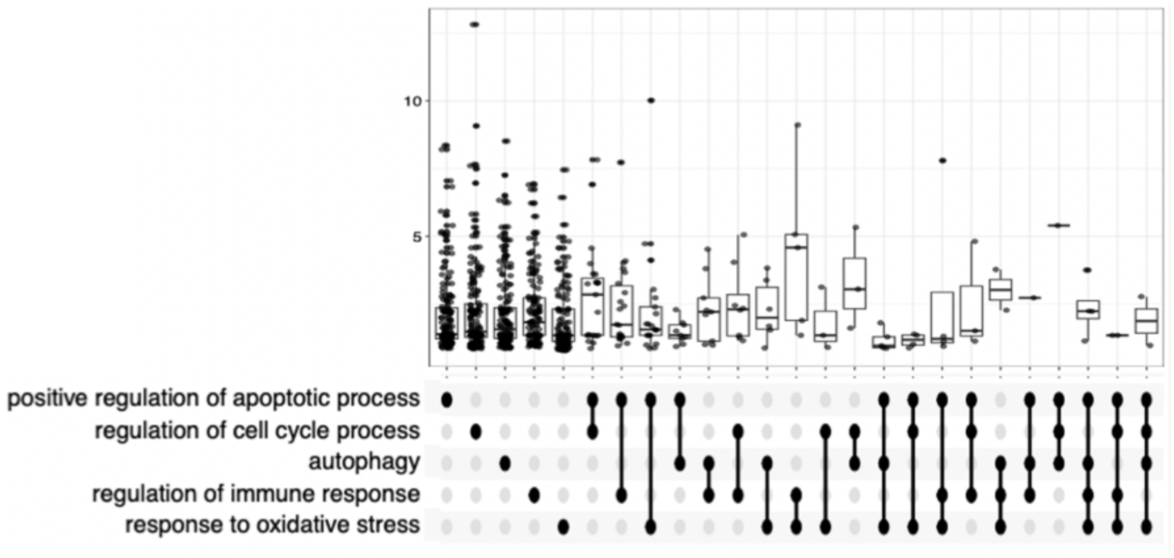

Figure S10. RNA-seq based pathway correlation analysis among oxidative stress, autophagy, apoptosis, and immune response related genes.

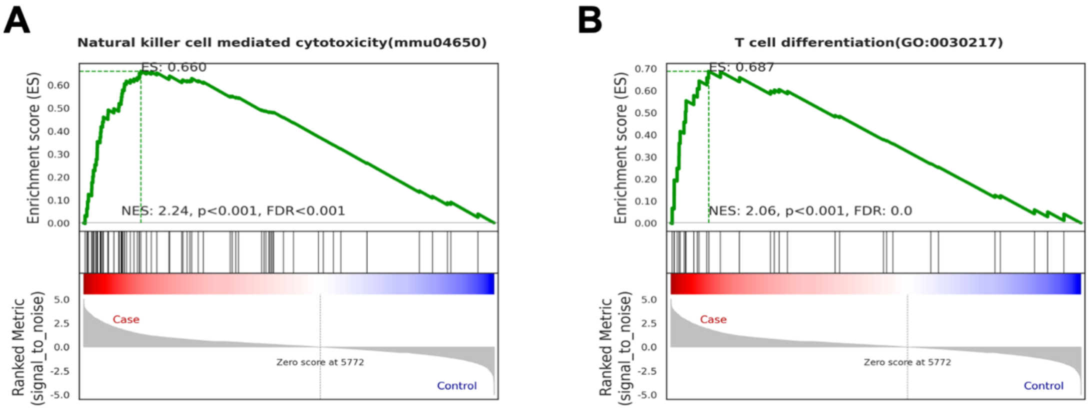

Figure S11. GSEA illustrating the activation of (A) natural killer cell-mediated cytotoxicity and (B) T cell differentiation pathways.

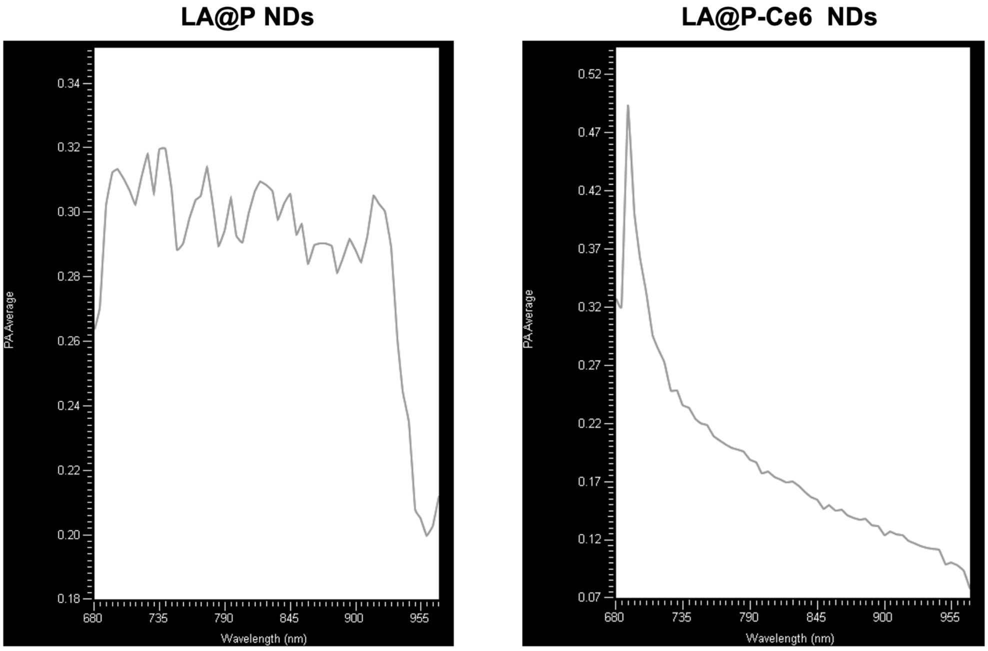

Figure S12. PA spectral scanning of LA@P NDs and LA@P-Ce6 NDs.

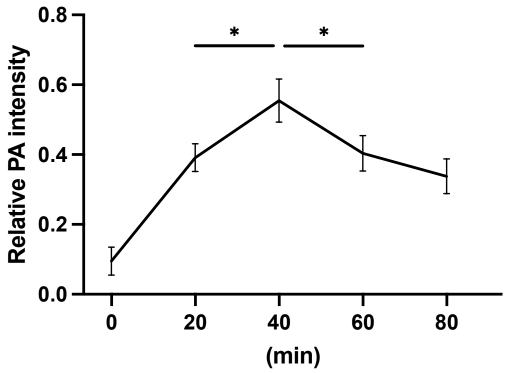

Figure S13. Relative PA intensity change after the injection of LA@P-Ce6 NDs.

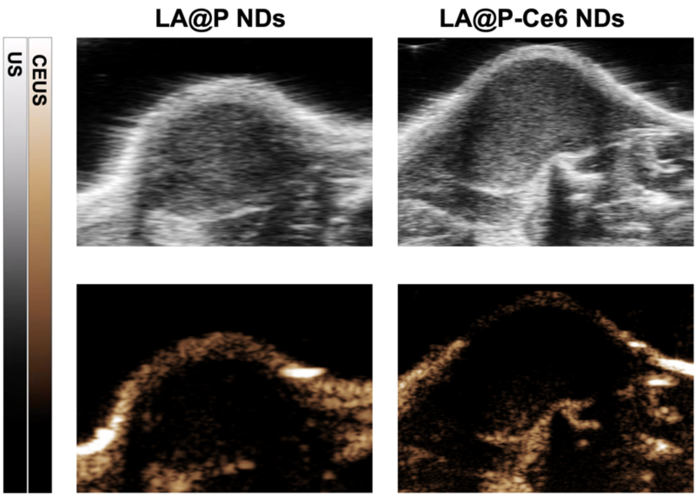

Figure S14. *In vivo* US and CEUS imaging at 80 min post-injection.

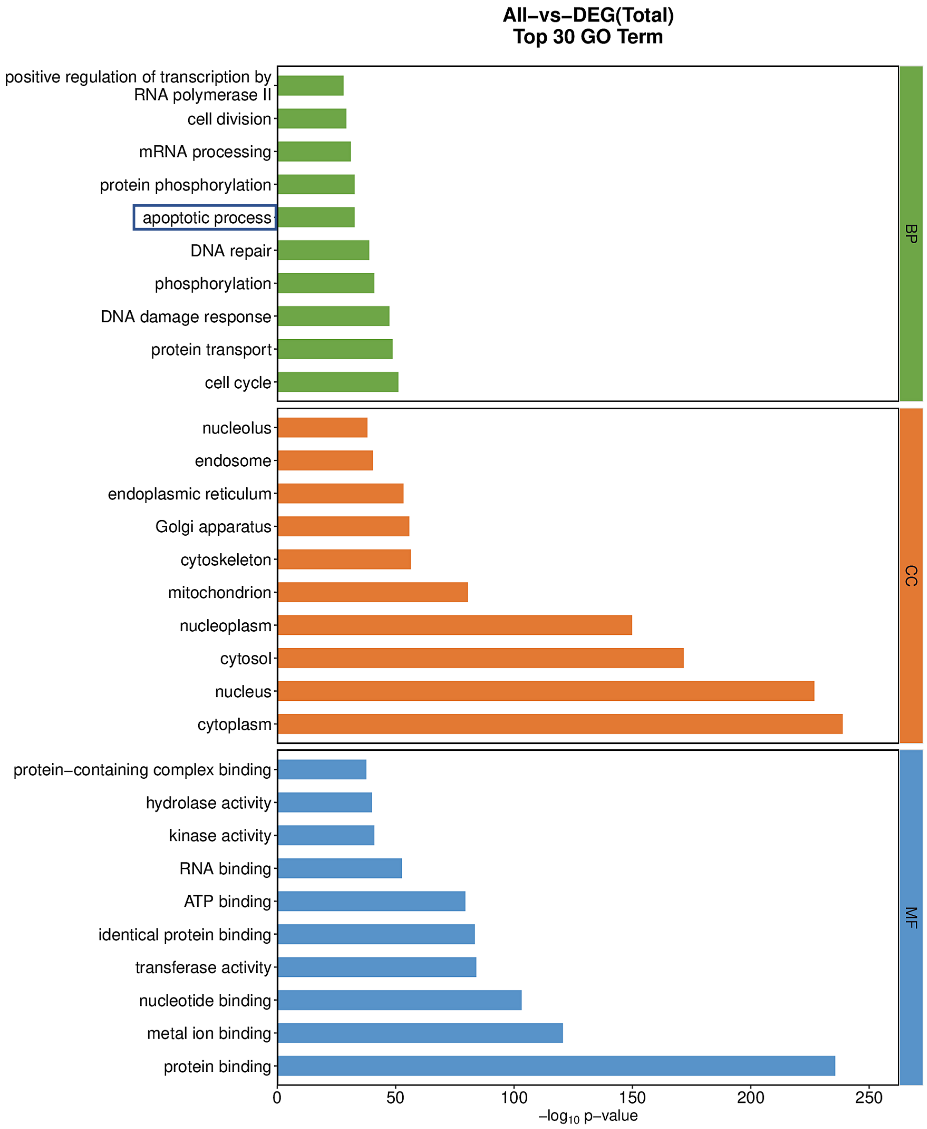

Figure S15. Top 30 GO terms of DEGs from RNA-seq analysis.

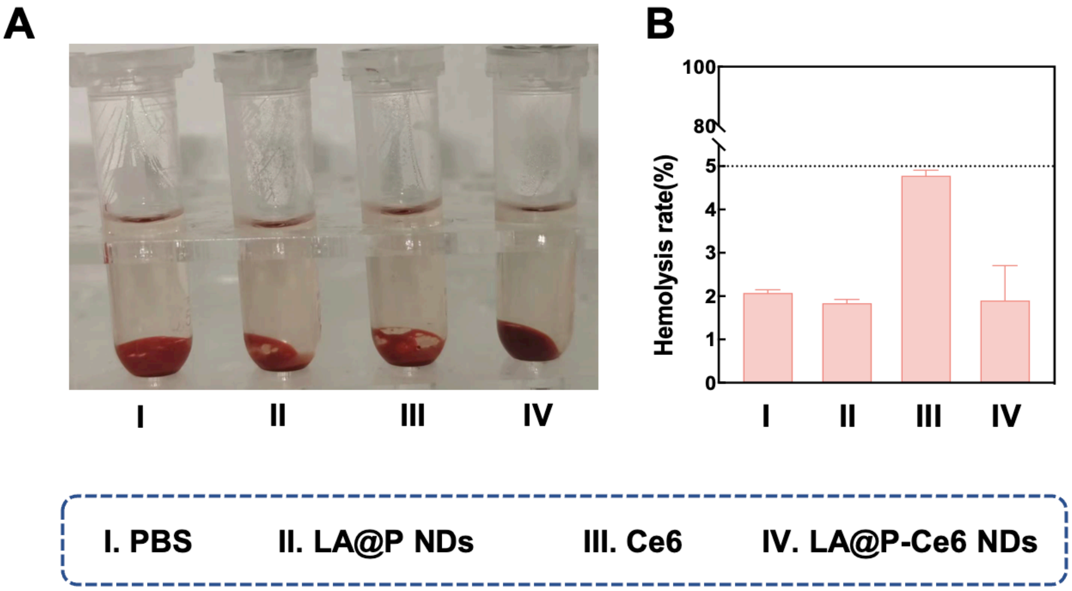

Figure S16. Assessment of blood compatibility of different treatments. (A) Photographs of red blood cells after 1 h incubation under different treatments. (B) Corresponding hemolysis rates. Data are presented as mean ± SD.

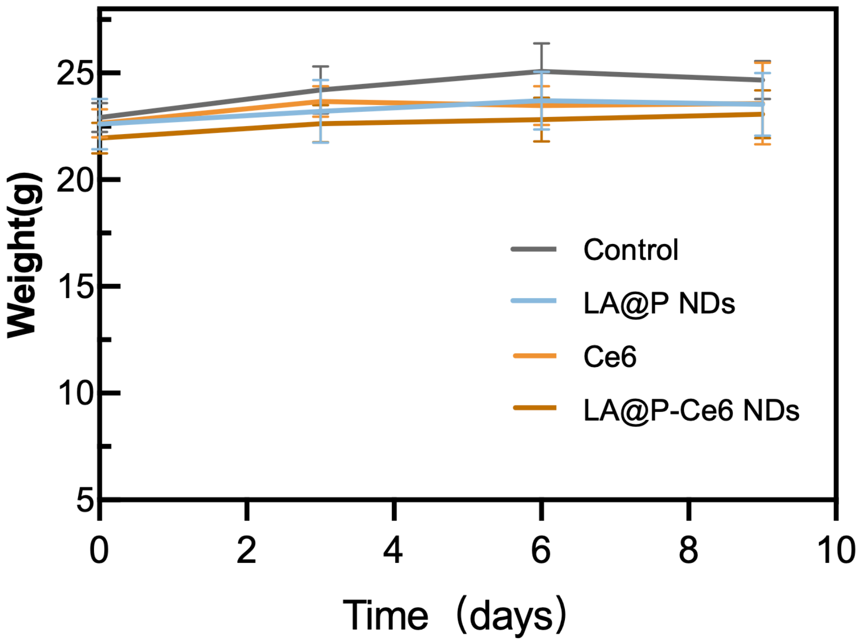

Figure S17. Body weight changes during treatments. Data are presented as mean ± SD. (n=4)

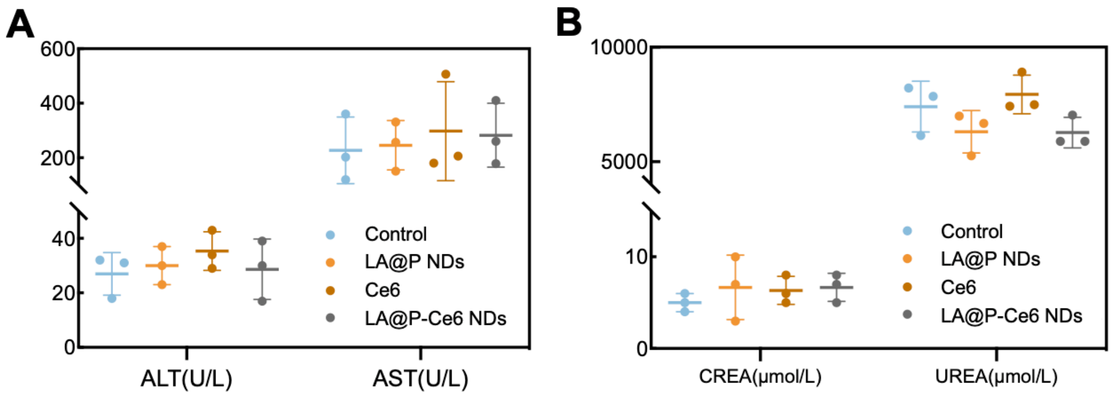

Figure S18. Assessment of hepatic and renal function after treatments. (A) Liver function assessed by serum ALT and AST levels. (B) Kidney function assessed by serum CREA and UREA levels. Data are presented as mean ± SD. (n=3)

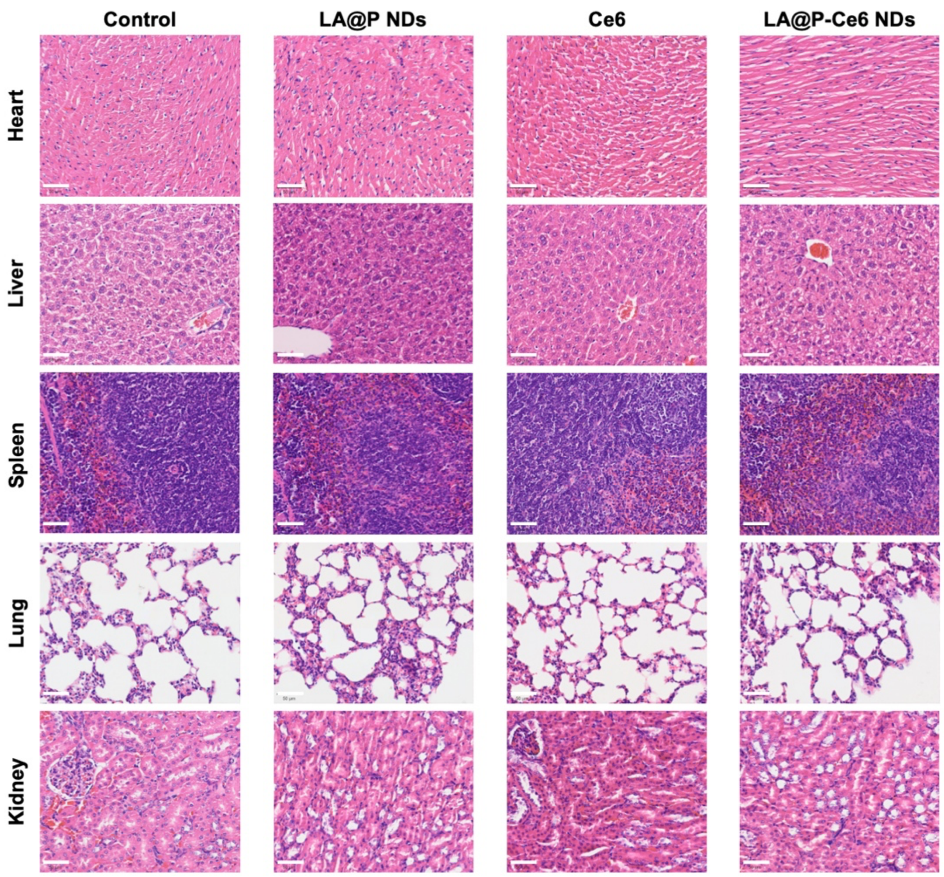

Figure S19. HE staining of major organs after the treatments. (Scale bar = 50 μm)

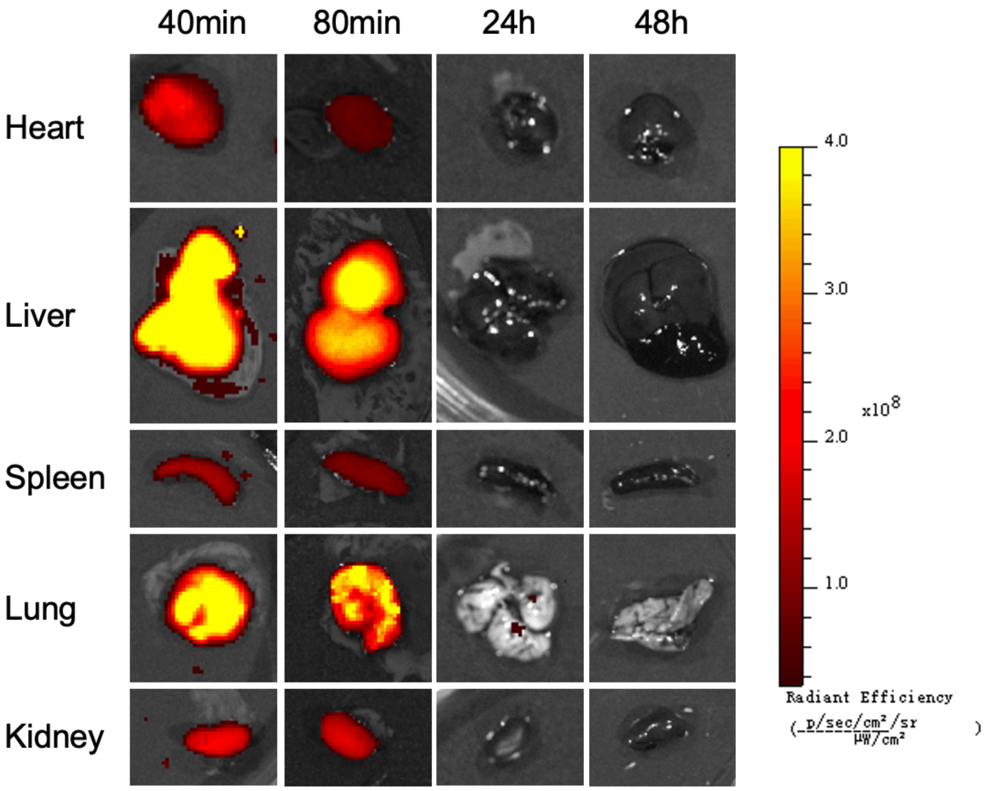

Figure S20. *In vivo* fluorescent and bioluminescent images of major organs after administration with LA@P-Ce6 NDs.
